# Optimization of a Resolution Process Allowing Access to Both Enantiomers of the Versatile Chiral Phosphine Ligand sSPhos on Scale

**DOI:** 10.1021/acs.oprd.6c00142

**Published:** 2026-06-25

**Authors:** Hamzah Sharif, Thomas D. Svejstrup, Staffan Karlsson, Robert J. Phipps

**Affiliations:** † Yusuf Hamied Department of Chemistry, 2152 University of Cambridge, Lensfield Road, Cambridge CB2 1EW, U.K.; ‡ Early Chemical Development, Pharmaceutical Sciences, R&D, AstraZeneca, Gothenburg 431 83, Sweden

**Keywords:** phosphine, resolution, atroposelective, sulfonation, sSPhos

## Abstract

Broadly effective, chiral monodentate phosphine ligands in asymmetric catalysis are relatively rare. sSPhos is a versatile, axially chiral phosphine that is capable of inducing high levels of enantioselectivity in a number of palladium-catalyzed transformations, including atroposelective Suzuki-Miyaura cross-coupling, arylative dearomatization, and carboetherification reactions. We have previously reported the chiral resolution of sSPhos by salt formation with quinidine, permitting the isolation of the *(R)* enantiomer. That process possessed several limitations, including relatively small scale, inability to access the *(S)* enantiomer, the requirement to presynthesize a trifluoroacetate salt of the dihydroquinine, and a maximum ee of 98% of *(R)*-sSPhos. In this work, we report further optimization and development of that procedure to permit it to be performed on a large scale. This simplified process permits the protonated, zwitterionic form of *(R)*-sSPhos to be obtained in >99% ee and *(S)*-sSPhos in 98% ee. The new modified procedure includes a resolution of racemic sSPhos by crystallization and was demonstrated on an 80 g scale to give access to both enantiomers of sSPhos in high ee and good yield. It is notable that the *(S)-*sSPhos enantiomer had previously been available only through preparative separation. We anticipate that this will assist in the incorporation of sSPhos as a chiral ligand in screening libraries and facilitate industrial application.

## Graphical abstract

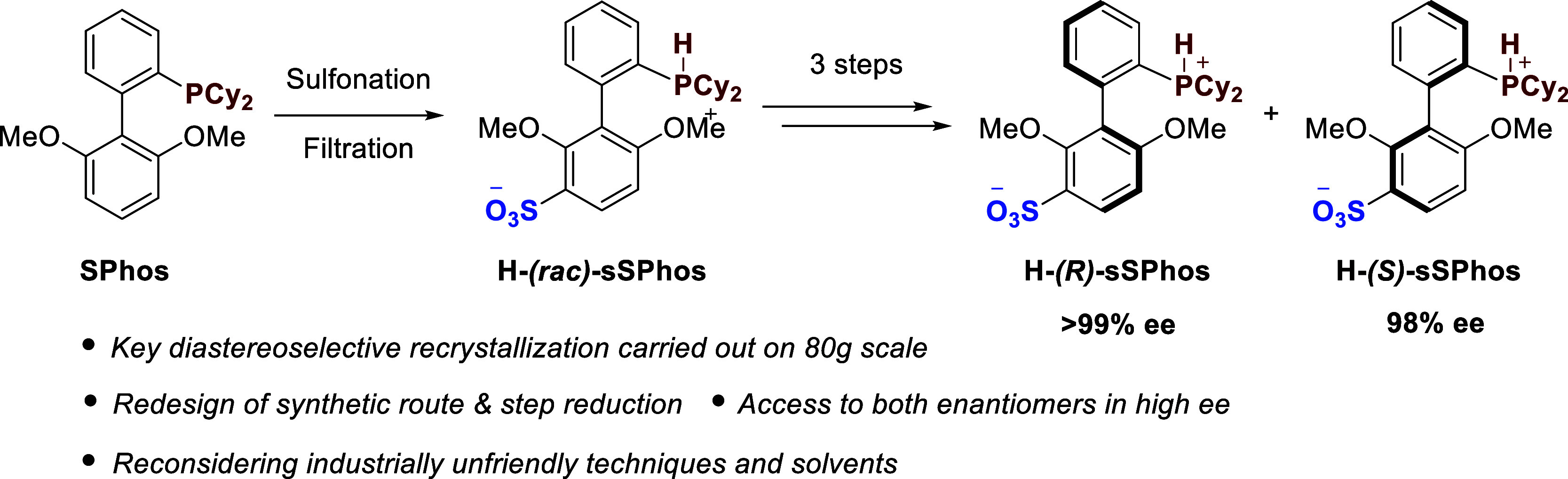

## Introduction

Palladium-catalyzed reactions are ubiquitous in the pharmaceutical industry, easing the formation of key carbon–carbon and carbon–heteroatom bonds in molecules of medicinal interest.^1^ The importance of such transformations is evident in modern drug synthesis, with Buchwald–Hartwig, Negishi, and Suzuki–Miyaura couplings all being frequently employed.^2^ For processes that generate stereochemistry, preparative chiral separation is sufficient for early-stage drug discovery, but enantioselective transformations are more time- and cost-beneficial when candidate progression requires scale-up and manufacture.^3^ Among the most commonly used ligands for palladium catalysis are the dialkyl-*ortho*-biaryl phosphines, developed by Buchwald.^4^ SPhos, first reported in 2004, has become one of the most widely used ligands for Suzuki–Miyaura cross-couplings.^5^ sSPhos is a sulfonated variant of SPhos that was developed as a water-soluble ligand for aqueous cross-coupling reactions and has been used extensively for that purpose (Figure 1, A).^6^ Since 2018, our laboratory has reported a number of studies that repurpose sSPhos as a bifunctional phosphine ligand; the sulfonate group is highly effective at hydrogen bonding and ionic interactions and can engage in noncovalent interactions with substrates and reactive intermediates in a variety of ways. This was first used for site-selective cross-coupling using (*rac*)-sSPhos to control selectivity in oxidative addition via an electrostatic interaction with the substrate.^7^ Realizing that sSPhos is actually a chiral ligand, we then resolved it and investigated its application in enantiopure form. Through various studies from our lab, it has been demonstrated to be effective in atroposelective Suzuki–Miyaura coupling,^8^ enantioselective arylative dearomatization,^9^ Tsuji–Trost reactions,^10^ and alkene oxyarylation and aminoarylation (Figure 1, B).^11^ We have discovered a sufficient range of enantioselective processes using this ligand to believe that it can be regarded as a broadly applicable chiral ligand of utility in the field.^12^ This is valuable because there are not a great deal of chiral monodentate phosphines available. For this reason, we sought to develop a scalable and economic resolution process to enable ready access to both enantiomers of sSPhos.

**Figure 1:**
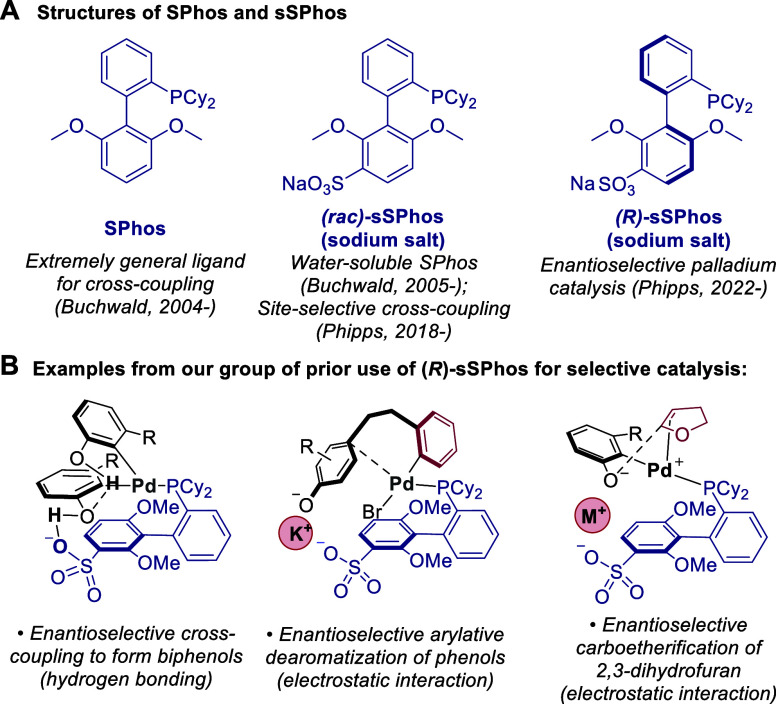
Outline of ligand structures and prior applications of sulfonated SPhos (sSPhos). **A** Structures of SPhos and sSPhos. **B** Examples from our group of prior use of (*R*)-sSPhos for selective catalysis.

In our first report, enantiopure sSPhos resolution was demonstrated by two means.^8a^ First, preparative SFC separation at AstraZeneca. Second, chiral resolution by diastereomeric salt formation followed by recrystallization, permitting isolation of the *(R)* enantiomer of sSPhos.^13^ This process consisted of six discrete steps, starting with SPhos (Scheme 1). Sulfonation using concentrated sulfuric acid, according to the literature-reported conditions^6a^ gives Na-(*rac*)-sSPhos after basic workup, and this was typically purified by column chromatography to remove traces of oxidized byproduct. Separately, the trifluoroacetic acid salt of quinidine is prepared, and this is subjected to ion exchange with the purified Na-(*rac*)-sSPhos. Following this, recrystallization of the 1:1 mixture of diastereomeric salts from MeCN yields a dr of 96:4 of QD*-(R)-*sSPhos. The original protocol called for hot filtration followed by a second MeCN recrystallization, which upgraded the dr to 99:1. Cation exchange using AmberLite IR120H resin, followed by a basic workup, yields the desired Na-*(R)-*sSPhos with 98% ee.

**Scheme 1:**
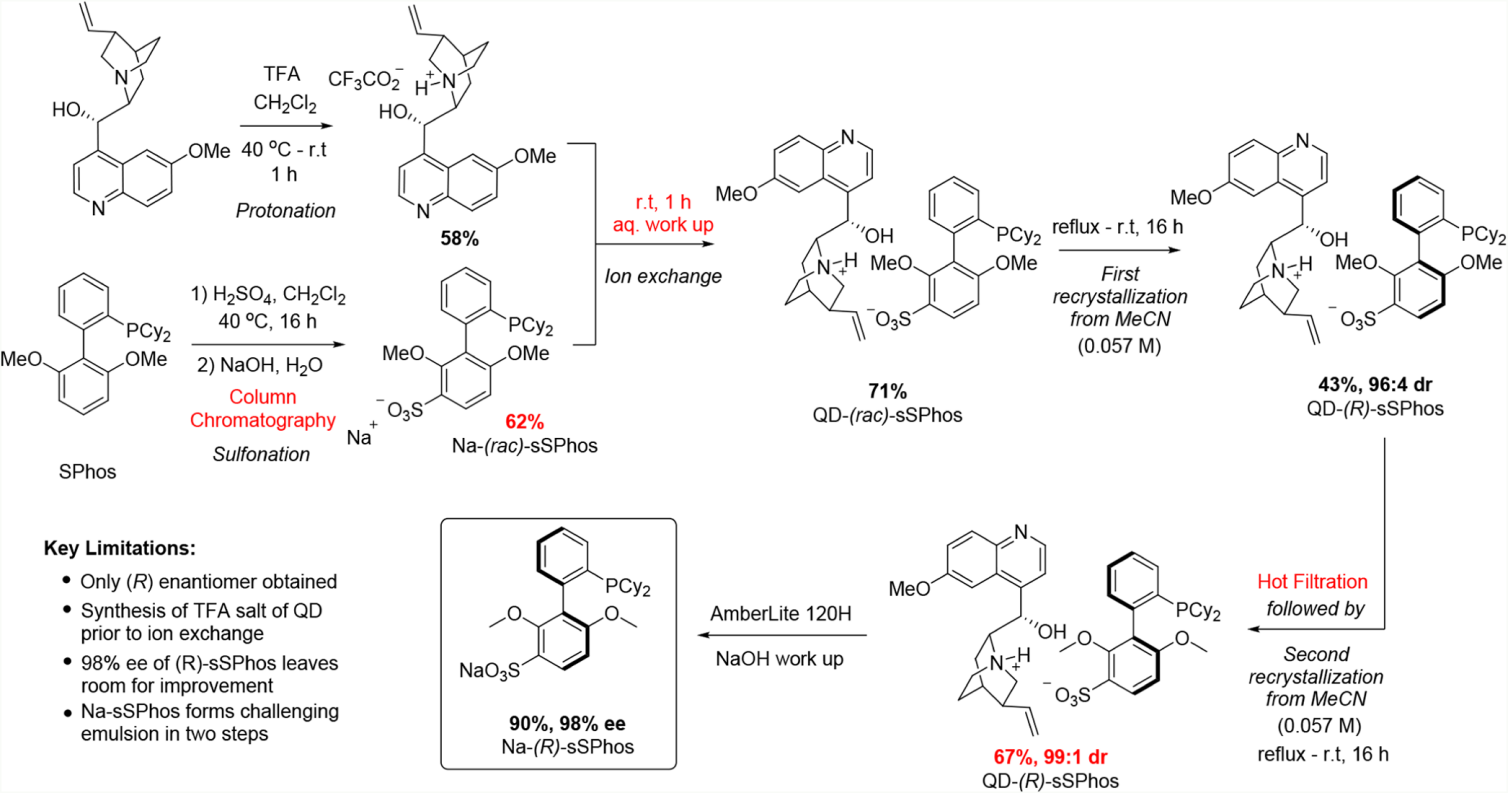
Prior Reported Synthetic Process for Synthesis and Resolution to Obtain Na-(*R*)-sSPhos by Recrystallization

This resolution procedure, which was previously reported, possessed a number of limitations, namely:

1. Access to the (*S*)-enantiomer of sSPhos was not demonstrated.
2. Presynthesis of the trifluoroacetic acid salt of dihydroquinine was required before ion exchange with Na-(*rac*)-sSPhos.
3. A maximum ee of 98% *(R)*-sSPhos was achieved after two successive recrystallizations from MeCN.
4. Two steps involve the isolation of Na-sSPhos, which forms a challenging emulsion that becomes problematic on scale.
5. Chromatography was used in the initial sulfonation step to ensure purity going into the recrystallization step.

At the outset of this study, we sought to overcome some of the limitations of this route using a process chemistry viewpoint, with the aim of facilitating the scale-up of the resolution of this valuable monodentate chiral phosphine. Herein, we report a streamlined, scaled-up process that permits access to the zwitterionic, protonated form of the ligand, H*-(R)*-sSPhos (>99% ee), on a large scale. Furthermore, we demonstrate that H*-(S)-*sSPhos can now be obtained from the resolution in 98% ee. We anticipate that this improved and scalable procedure for the resolution will encourage wider incorporation of sSPhos into ligand libraries in industry and academia.

## Results and Discussion

The first step we sought to optimize to make it amenable to larger scale was the sulfonation of SPhos.^6a,6c^ Previously, we had found that it was important for the subsequent resolution step that the material be very pure and, therefore, used column chromatography to purify Na*-(rac)-*sSPhos. Here, we sought an alternative purification protocol and also aimed to remove the basic workup required to obtain the sodium salt. The latter operation results in a challenging emulsion due to the surfactant-like properties of Na-sSPhos, which requires extensive extraction with CH_2_Cl_2_. Interestingly, protonation of Na-sSPhos, to give H-sSPhos, results in protonation at the phosphorus atom to give a zwitterionic compound with a ^31^P NMR resonance appearing as a broad peak in the 15–20 ppm range, compared to the sharp singlet at ∼−9 ppm for the unprotonated phosphine of Na-sSPhos. The solubility of this zwitterionic compound differs drastically from the sodium salt, with the protonated form being soluble only in very polar solvents such as MeOH. We hypothesized that we could leverage this insolubility to allow isolation of H*-(rac)-*sSPhos by precipitation after sulfonation. If successful, this would avoid the chromatographic purification, the aqueous extraction step, and open the possibility of also removing the next step of ion exchange with the quinidinium trifluoroacetate salt.

We evaluated sulfonation conditions with attempted isolation of the zwitterion. Removing the originally used CH_2_Cl_2_ solvent (Table 1, entry 1) would be advantageous on a large scale, and use instead of an excess of conc. H_2_SO_4_ gave quantitative conversion to H*-(rac)-*sSPhos at 40 °C (entry 2). Performing this at room temperature (entry 3) and with a smaller excess of acid (entry 4) also gave quantitative conversion, although this suffered upon reducing the excess further to three equivalents (entry 5). Unfortunately, for entries 2–4, attempted isolation of the product via precipitation by addition of the antisolvent EtOAc resulted in a sticky white solid, which still contained water and H_2_SO_4_ that could not be completely removed. This interfered with the subsequent salt formation and diastereoselective recrystallization, and led us to seek an alternative protocol.

**Table 1:**
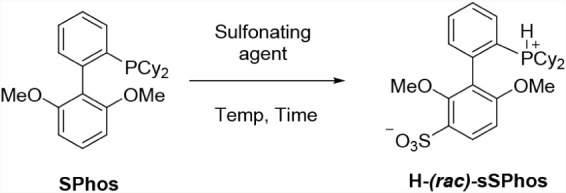
Initial Sulfonation Attempts

Entry	Sulfonating agent	Temp	Time(h)	Conversion by LCMS^a^
1	H_2_SO_4_ (1.2 equiv) in DCM	40 °C	17	63%
2	Conc. H_2_SO_4_ (26 equiv)	40 °C	17	Quant
3	Conc. H_2_SO_4_ (26 equiv)	r.t	17	Quant
4	Conc. H_2_SO_4_ (13 equiv)	r.t	17	Quant
5	Conc. H_2_SO_4_ (3 equiv)	r.t	17	43%

a Product conversion determined by peak distribution in **LCMS**.

We have recently explored the use of an “activated” sulfonating agent generated by the combination of trifluoroacetic anhydride (TFAA) and a small excess, relative to the substrate, of conc. sulfuric acid.^14^ This was found to be a reactive sulfonating agent for RuPhos, but comparatively mild in that large excesses of strong acid are not required.^8b^ Additionally, the trifluoroacetic anhydride removes water, generating an anhydrous sulfonating agent. Utilizing these sulfonation conditions resulted in quantitative sulfonation after 17 h, and 95% isolated yield could be obtained by precipitation of the zwitterion as a fine white solid, which presented no purification issues using this protocol (Table 2 entry 1).^15^ Despite these encouraging results using trifluoroacetic acid as a solvent, for environmental reasons, we searched for alternative nonfluorinated acids (acetic acid and ethyl acetate were evaluated) but found that lower yields and undesired oxidation of SPhos occurred (entries 2 and 3). Similarly, we evaluated alternative nonfluorinated anhydrides but found that no sulfonation of SPhos was observed (entries 4–6). Scaling up the successful procedure to 10 mmol and then 50 mmol in a round-bottomed-flask gave essentially the same result as the 1 mmol scale (entries 7 and 8 vs entry 4). A further scale-up to 250 mmol using a Radleys reactor resulted in consistent total isolated yield, following two successive precipitations, demonstrating the viability of this protocol for rapid access to large amounts of the racemic sSPhos zwitterion (entry 9).

**Table 2:**
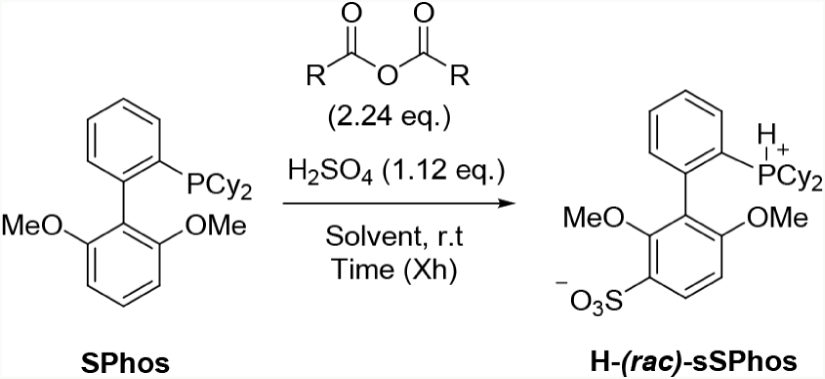
Sulfonation Using Activated H_2_SO_4_ Reagents

Entry	R	Solvent	Time (h)	Conversion by LCMS^a^	Isolated Yield^b^	Scale (mmol)
1	CF_3_	TFA	17	quant	95%	1
2	CF_3_	Acetic acid	17	67%	-	1
3	CF_3_	EtOAc	40	67%	-	1
4	Me	TFA	17	0%	-	1
5	^t^Bu	TFA	17	0%	-	1
6	Ph	TFA	17	0%	-	1
7	CF_3_	TFA	17	quant	95%	10
8	CF_3_	TFA	17	quant	98%	50
9^c^	CF_3_	TFA	17	quant	65% + 30%^d^	250

a Product conversion determined by peak distribution in **LCMS**.

b Isolated by trituration with EtOAc.

c Using a Radleys reactor.

d 65% isolated yield obtained after the first trituration and further 30% obtained after the second trituration.

With access to H*-(rac)*-sSPhos now forthcoming, optimization of the diastereoselective recrystallization was performed. In the previous method, where the sodium salt of sSPhos was used, the sodium cation was exchanged with protonated quinidine from its TFA salt. This necessitated a separate prior step to obtain the latter. Furthermore, the use of CH_2_Cl_2_ in the ion exchange was problematic because it was challenging to remove all traces of CH_2_Cl_2_ from the resulting diastereomeric salt, which impacted the effectiveness of the subsequent recrystallization step in MeCN. We saw the opportunity here to streamline and improve this process by simply deprotonating the zwitterion with quinidine to form the diastereomeric salts *in situ*. This was found to be straightforward—the chiral amine and H*-(rac)*-sSPhos were heated in MeCN until the solution was homogeneous, and then it was allowed to cool and crystallize out. Gratifyingly, using quinidine, this simplified protocol gave the same outcome (96:04 d.r.) from the first recrystallization as had been observed using the prior, more complicated protocol (Table 3, entry 1). At this point, we decided to explore a small panel of alternative chiral bases, as these had not been rigorously evaluated under the final recrystallization conditions in the earlier study. Interestingly, substituting quinidine (QD) with quinine (QN) or cinchonidine (CD) resulted in almost no crystallization occurring on cooling (entries 2 and 3). With cinchonine (CN), a 12% yield was obtained, but this was found to be completely nonselective (entry 4). l-Proline amide (A1) did not give a homogeneous solution even after extended heating (entry 5). Two other commonly utilized chiral bases also gave no yield (entries 6 and 7). This exploration underlines the unique utility of quinidine for this resolution, in combination with acetonitrile. Using quinidine, further evaluation of solvents and concentrations was carried out, but this resulted in no significant improvements in the d.r. after a single recrystallization (see Supporting Information for full details).

**Table 3:**
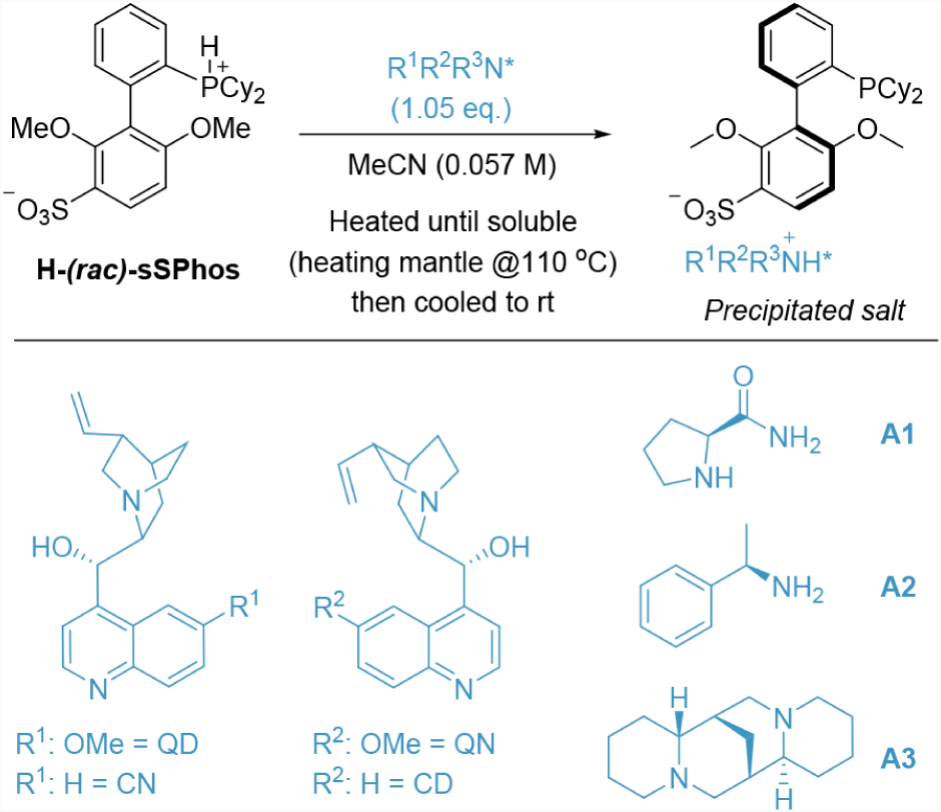
Evaluation of Chiral Bases for Diastereoselective Recrystallization of H*-(rac)-*sSphos

Entry	Chiral Base	Isolated Yield	d.r^b^
1	QD	42%	96:04
2	QN	<5%	-
3	CD	<5%	-
4	CN	12%	*rac*
5^a^	A1	-	-
6	A2	<5%	-
7	A3	<5%	-

a Not fully soluble after heating.

b Deduced based on the ee of H-sSPhos, after cation exchange, as analyzed by chiral SFC.

As a final effort to avoid a second recrystallization, a temperature-solubility analysis of QD-*(R)-*sSPhos and QD-*(S)-*sSPhos was performed in hopes of improving diastereoselectivity by modulation of the end temperature (Chart [Fig chart1]). While a slight improvement in selectivity was obtained by setting the end temperature to 40 °C, an unexpected and significant drop in yield occurred (Table 4, entry 2 vs entry 1). This could be improved somewhat by leaving it for a longer time, but this resulted in lower diastereoselectivity and still unacceptably low yield (entry 3). Thus, room temperature (20–23 °C) was retained for the end of the recrystallization for the scaling-up evaluations. A 9.5 mmol scale using standard laboratory equipment yielded exactly the same results as the 1 mmol (entry 4). Fortunately, this method was found to be reproducible for the 49 and 170 times scale-up experiments using the Radleys reactor, with only the 170 mmol scale giving a slight decrease in yield but retaining the 96:04 d.r. (entries 5 and 6).

**1 chart1:**
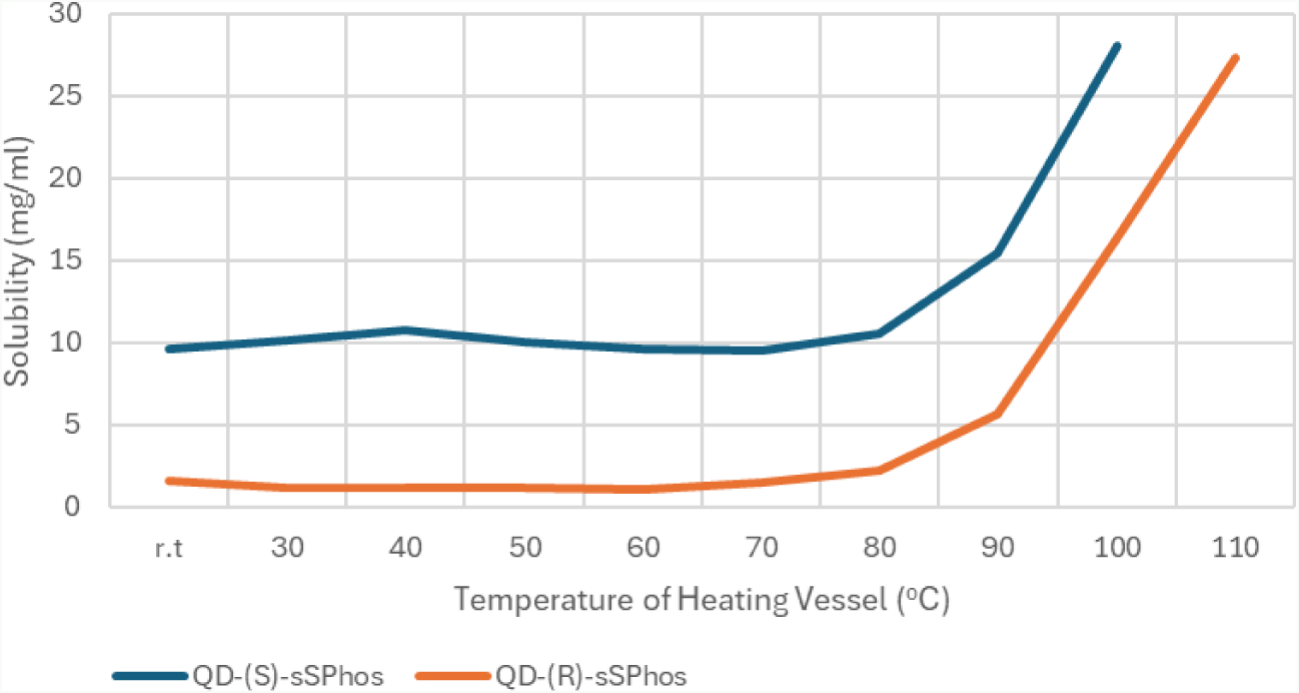
Temperature-Solubility Curve for QD-(*R*)-sSPhos and QD-(*S*)-sSPhos

**Table 4:**
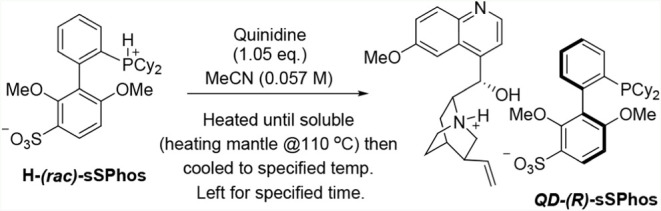
Evaluation of Recrystallization End Temperature and Time

Entry	End T (°C)	Time (h)	Isolated Yield	d.r^a^	Scale (mmol)
1	r.t	17	42%	96:04	1
2	40	17	10%	97:03	1
3	40	30	20%	92:08	1
4	r.t	17	42%	96:04	9.5
5^b^	r.t	17	43%	96:04	49
6^b^	r.t	17	39%	96:04	170

a Deduced based on the ee of H-sSPhos after cation exchange, as analyzed by chiral SFC.

b Using a Radleys reactor.

With the scalability of the first recrystallization established, the second recrystallization was next carried out to upgrade d.r. In the prior work, a hot filtration was performed during the second recrystallization as small amounts of unidentified insoluble material were observed when heating. As performing hot filtrations on a larger scale is very challenging, we wished to dispense with this for scale-up and so increased the volume of MeCN used in the hope that the hot filtration might not be needed. Fortunately, this was successful and still resulted in very high d.r. with good yield (Table 5, entry 1). On sequential scale-up, this was found to be reproducible, with the final two entries being performed in the Radleys reactor (entries 2–4). The single-crystal X-ray structure had been determined on this material in our previous study (CCDC 2171203).^8a^ XRPD analysis is provided in the Supporting Information.

**Table 5:**
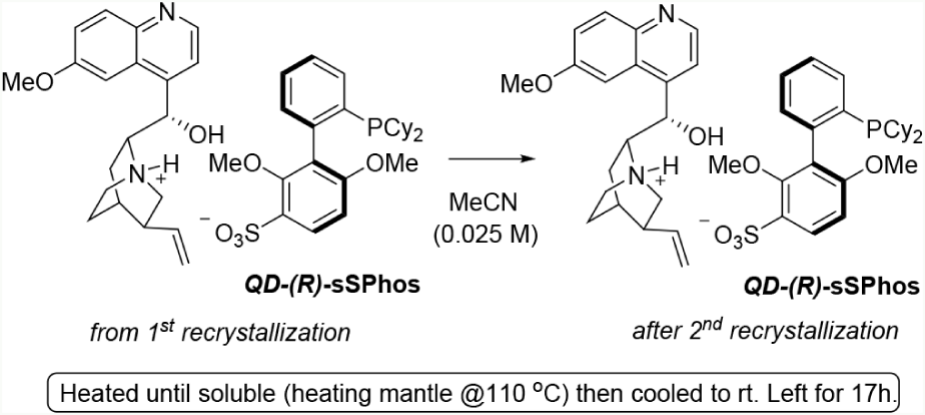
Second Recrystallization of QD-(*R*)-sSphos

Entry	Yield	d.r.^a^	Scale (mmol)
1	80%	>99:01	0.42
2	81%	>99:01	3.99
3^b^	81%	>99:01	21.1
4^b^	80%	>99:01	63.2

a Deduced based on the e.e., of H-sSPhos, after cation exchange, as analyzed by chiral SFC.

b Using a Radleys reactor.

Finally, AmberLite IR120H, a strongly acidic exchange resin, was used to remove the chiral cation and liberate H*-(R)-*sSPhos.^16^ The use of 30 g of AmberLite per gram of QD-*(R)-*sSPhos was found to give complete protonation, and this was carried out on batches of 2 g and 10 g without issue (Scheme 2). The ee of the material was determined to be >99% by chiral SFC analysis; the minor enantiomer was not visible on the trace.

**Scheme 2:**
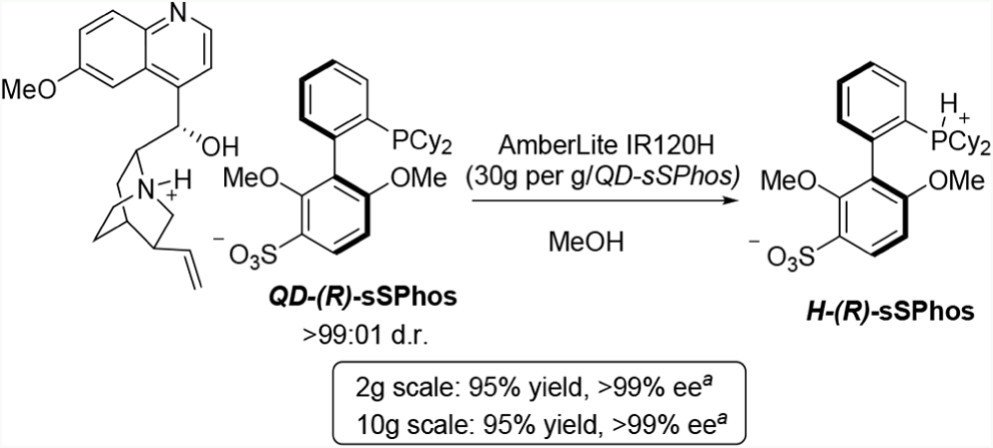
Removal of Chiral Cation using AmberLite^a^

a ee determined by chiral SFC analysis.

With a scalable route to access the *R* enantiomer established, investigation into accessing the *S* enantiomer was pursued. We had originally hoped that the use of quinine (QN), the pseudoenantiomer of quinidine (QD), may allow this, but from the chiral base screen previously performed with H*-(rac)*-sSPhos, QN and most other chiral bases yielded very little yield in MeCN. As such, effort was focused on enriching the QD-*(S)*-sSPhos salt that remained in the filtrate after the first recrystallization. Analysis showed that the filtrate consistently contained QD*-(S)-*sSPhos with a 15:85 d.r on 1, 9.5, and 49 mmol scales (Table 6, entries 1–3) and a slightly lower 18:82 d.r for the 170 mmol example (entry 4).

**Table 6:**
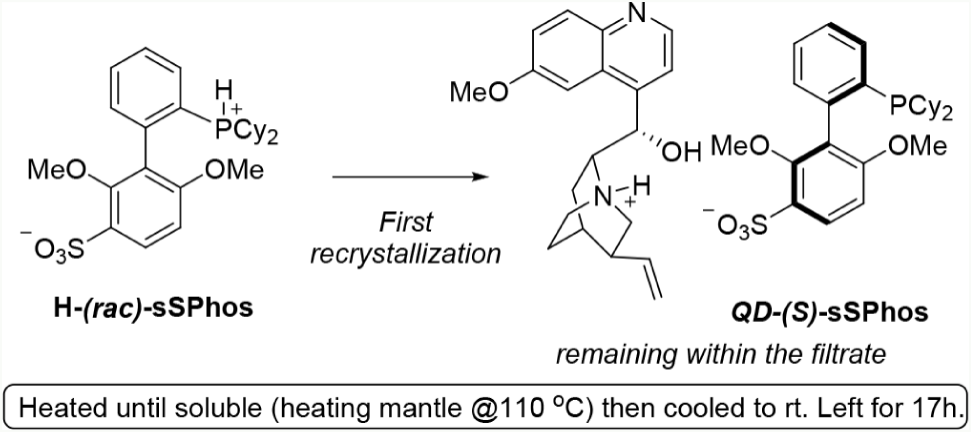
Analysis of Diastereomeric Enrichment of the Filtrate from the First Recrystallization Cycle

Entry	Yield	d.r^a^	Scale (mmol)
1	56%	15:85	1
2	58%	16:84	9.5
3^b^	55%	15:85	49
4^b^	60%	18:82	170

a Deduced based on the e.e., of H-sSPhos, after cation exchange, as analyzed by chiral SFC.

b Using a Radleys reactor.

We next decided to remove the chiral cation to liberate enantioenriched H-(*S*)-sSPhos, with the original goal of attempting recrystallization on this material to further enrich its optical purity. As shown in Scheme 2, if 30 g of AmberLite IR120H resin per g/QD-sSPhos is used, complete protonation is obtained to give a 95% yield of H-sSPhos with the expected ee of 64% based on a dr of 18:82 (Table 7, entry 1). We discovered adventitiously that if we used less AmberLite resin (15 g resin per g/QD-sSPhos instead of 30 g), we appeared to obtain incomplete protonation, as might be expected. Intriguingly, the liberated H-sSPhos was enriched in the (*S*)-enantiomer and could be readily separated from the remaining QD-sSPhos in a subsequent filtration step due to the poor solubility of H-sSPhos in several solvents. This fortuitous outcome suggested that the (*S*)-containing diastereomer of QD-sSPhos may be preferentially protonated on the resin to form a zwitterion. We initially evaluated this using EtOAc as the filtration solvent, in which H-sSPhos is insoluble. As expected, the yield of H-sSPhos was reduced, but an ee of 82% could be obtained, a substantial improvement (entry 2 vs entry 1). By switching the filtration solvent to MeCN, the ee could be increased to 98% (entry 3), and the process could be reproducibly scaled up; starting from 10 g of QD-sSPhos gave a 40% yield of H-(*S*)-sSPhos with 98% ee (entry 4).

**Table 7:**
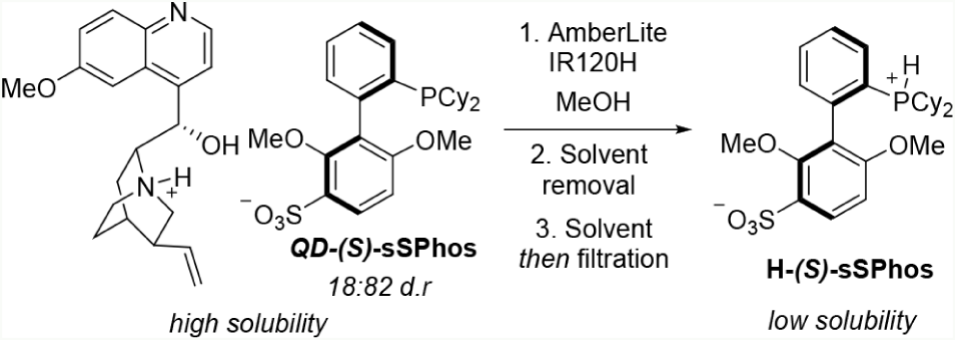
Incomplete Protonation Using AmberLite Followed by Filtration

Entry	g AmberLite/g starting material	Filtration Solvent	Yield	e.e. (%)^a^	Scale (g of starting material)
1	30	----	95%	64	2
2	15	EtOAc	40%	82	2
3	15	MeCN	38%	98	2
4	15	MeCN	40%	98	10

a ee determined by chiral SFC analysis.

Intrigued by this observation, we evaluated standard Bronsted acids in solution, rather than AmberLite, for the protonation (Table 8). We found that by following an analogous protocol, we reliably obtained 98% ee of the liberated H-(*S*)-sSPhos using several common acids, including HCl, TFA, and pTsOH (entries 1–3). In all three cases, similar yields were obtained compared with when AmberLite was used (Table 7). We therefore experimented with higher equivalents of acid than 0.6 to test whether yield could be increased without compromising ee in the precipitated H-(*S*)-sSPhos. This was found to be possible—2.4 equiv of pTsOH gave 62% yield, still with 98% ee (entry 5). Attempting a higher amount at 10 equiv precluded precipitation from MeCN at the end of the reaction, but we have not carried out further optimization. We also evaluated the use of pTsOH to protonate the QD*-(R)-*sSPhos salt obtained in >99:1 d.r. from the second recrystallization. This worked very well, providing an alternative approach for isolation of H*-(R)-*sSPhos if AmberLite is deemed undesirable (entry 7). Additionally, our standard protocol used sonication as part of the precipitation process from MeCN, but we found that this was not necessary and omitting sonication did not adversely impact yield (entry 8). We did consider whether racemic H-sSPhos may have higher solubility in MeCN than a single enantiomer, this being responsible for the enrichment. A brief solubility comparison showed both had very low solubility, although the racemate was slightly more soluble at room temperature (0.4 mg/mL vs 0.07 mg/mL, see SI for details). To test the possibility that enantioenrichment was purely a result of the solubility difference between H*-(S)-*sSPhos and H*-(rac)-*sSPHos, we attempted to enrich the isolated H-sSPhos (18:82 d.r.) by simply adding MeCN, sonicating, and filtering, but this yielded no enrichment (see SI). Although the precise reason for the enrichment of H-(*S*)-sSPhos remains unclear, the protocol has been demonstrated to be robust and a very effective way of obtaining both enantiomers of the ligand in high ee from this recrystallization protocol.

**Table 8:**
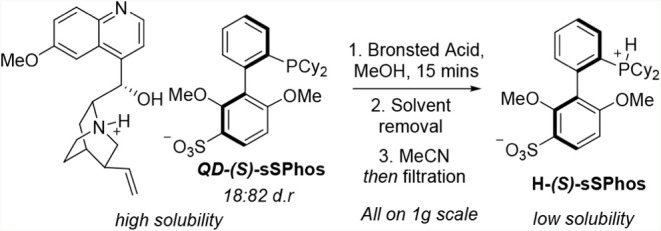
Incomplete Protonation Using Solution Bronsted Acids Followed by Filtration

Entry	Bronsted Acid	Yield	e.e. (%)^a^
1	HCl/Dioxane, 0.6 eq.	40%	98
2	TFA, 0.6 eq.	37%	98
3	pTsOH, 0.6 eq.	45%	98
4	pTsOH, 1.2 eq.	56%	98
5	pTsOH, 2.4 eq.	62%	98
6	pTsOH, 10 eq.	0%^b^	–
7^c^	pTsOH, 2.4 eq.	78%	>-99
8^d^	pTsOH, 2.4 eq.	60%	98

a ee determined by chiral SFC analysis.

b No precipitate was obtained.

c Using QD*-(R)-*sSPhos (>99:1) instead of QD*-(S)-*sSPhos (82:18).

d Without sonication.

At this stage, we demonstrated access to H-(*R*)-sSPhos with >99% ee and H-(*S*)-sSPhos with 98% ee, both on a multigram scale. However, all of our previous asymmetric catalysis methodology had utilized the sodium salt Na-(*R*)-sSPhos, rather than the zwitterion that we are now producing. One option would be to convert the zwitterion to the sodium salt by washing with aqueous sodium base. As mentioned previously, repeated extraction with CH_2_Cl_2_ permits isolation of Na-sSPhos but with the drawbacks of a challenging emulsion and large volumes of solvent required. Given that all our methodology reactions using Na-sSPhos employ inorganic bases in superstoichiometric amounts, we envisaged that it should be viable to add the zwitterion instead of the sodium salt, and it would be deprotonated by the excess base present, freeing up the phosphine atom for ligation to palladium. We tested this in the atroposelective Suzuki–Miyaura coupling of **1** and **2** to form 2,2’-biphenol **3**.^8a^ We performed a direct comparison of previously obtained Na-(*R*)-sSPhos (from preparative SFC separation) and H-(*R*)-sSPhos (obtained using the above protocol) on a screening scale of 0.1 mmol. Pleasingly, virtually identical results were obtained (Table 9, entries 1 and 2). We then demonstrated this was similarly effective on a 1 mmol scale (entry 3) and showed the effectiveness of H-(*S*)-sSPhos in delivering the opposite product enantiomer (entry 4). We were curious to evaluate QD-*(R)*-sSPhos as the excess base present should form Na*-(R)-*sSPhos *in situ,* with the main question being whether the presence of the liberated QD would impact the reaction. In the event, the Suzuki coupling proceeded smoothly, and the ee was even slightly improved to 96% ee (entry 5).

**Table 9:**
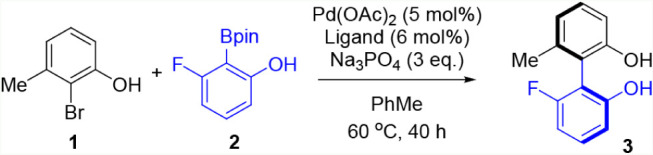
Ligand Comparison in Atroposelective Suzuki–Miyaura Cross-Coupling to Form 2,2’biphenol

Entry	Ligand	Yield	Ee (%)	Scale (mmol)
1	Na*-(R)-*sSPhos	82%	94	0.1
2	H-*(R)-*sSPhos	81%	94	0.1
3	H-*(R)-*sSPhos	75%	94	1
4	H-*(S)-*sSPhos	73%	–94	1
5	QD-*(R)-*sSPhos	68%	96	0.1

## Conclusions

In summary, we have extensively improved and extended the previously published protocol for the resolution of racemic sSPhos by salt formation with quinidine (Scheme 3). These modifications have been made with the intention of facilitating scaled-up synthesis and resolution of the ligand, starting from the cheaply available SPhos. Our modifications have overcome a number of challenges with the original synthesis, namely:

1. Access to the (*S*)-enantiomer was not previously demonstrated, but now this can be obtained in 98% ee by further manipulation of the filtrate from the first recrystallization. This was enabled by the discovery that the enriched QD salt can be selectively deprotonated using controlled quantities of AmberLite IR120H resin.
2. Presynthesis of the trifluoroacetic acid salt of dihydroquinine was previously required before ion exchange with r*ac*-sSPhos. This has been avoided by forming the zwitterion H*-(rac)*-sSPhos after sulfonation and then simply deprotonating this by using quinidine. Avoiding the use of the Na salt of sSPhos also simplified isolation following the sulfonation step.
3. A maximum ee of 98% (*R*)-sSPhos was achieved after two successive recrystallizations from MeCN in the prior protocol. Here, by adjusting the amount of solvent, >99% ee can be obtained after two recrystallizations, and this was shown to be viable on a large (51 g) scale.
4. Formation of the sodium salt Na-sSPhos involves a challenging emulsion that becomes problematic on a larger scale and was a feature of the previous protocol at several points (first step and final step). In the improved route, it is replaced in both places with the zwitterionic H-sSPhos. This can be precipitated out readily and does not involve an aqueous extraction. We show that this zwitterionic, protonated phosphine is just as effective as the analogous sodium in an enantioselective Suzuki–Miyaura coupling. The presence of an excess base in the reaction mixture ensures deprotonation to form the active ligand.
5. There is now no chromatography at any stage of the process.

**Scheme 3:**
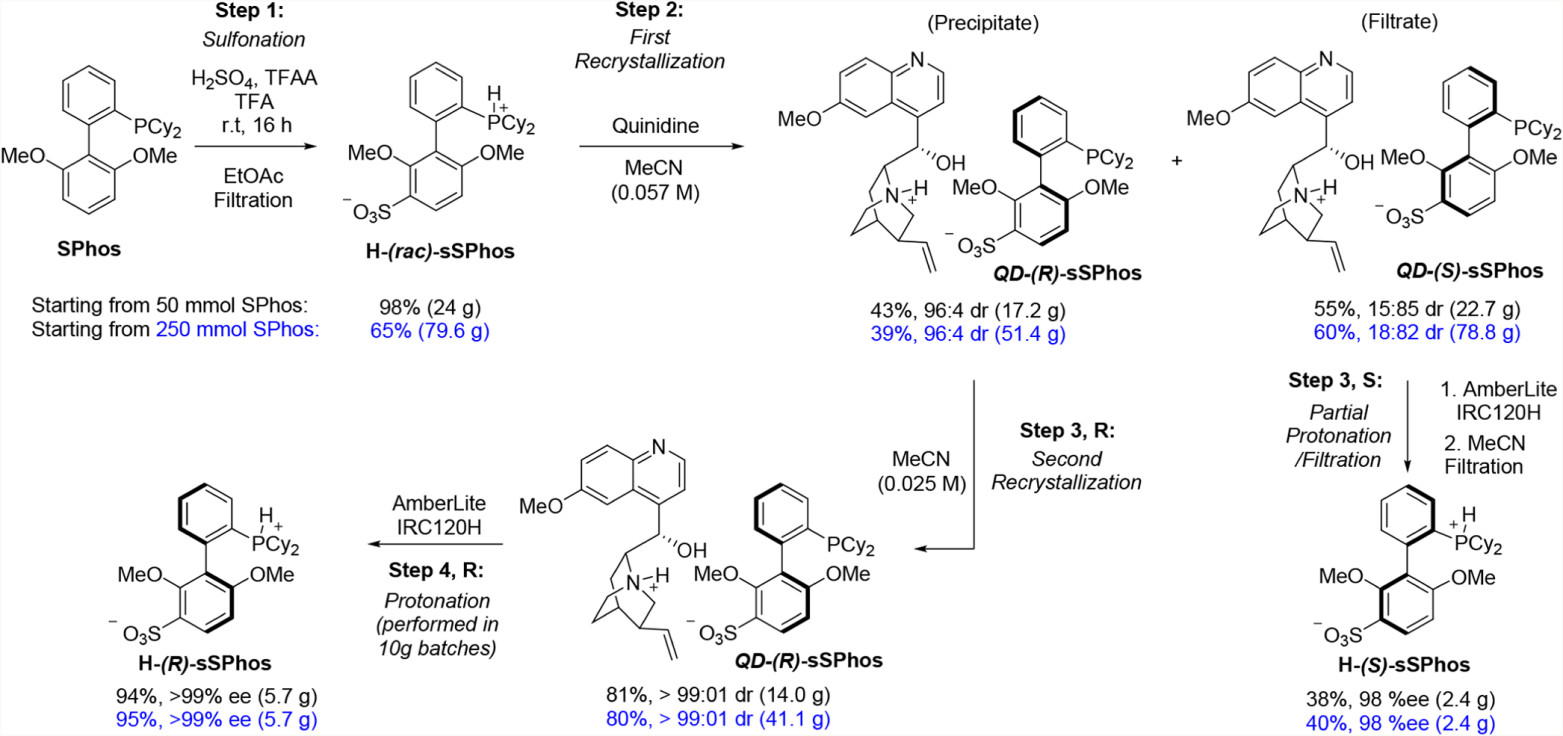
Summary of Improved Synthetic Process for Synthesis and Resolution of sSPhos to Obtain both Enantiomers on Scale

Combining all the above, the scale of the recrystallization has been demonstrated in a scale-up in a Radleys reactor, which commences with 102.7 g of SPhos and yields 41.1 g of >99:1 d.r. QD salt after two recrystallizations. This salt is taken through to the final zwitterionic ligand in 10 g batches as needed. The synthesis redesign achieves higher enantiopurity and yield by reducing the number of transformations, avoiding scale-up incompatible purifications, while also giving access to the *(S)*-sSPhos enantiomer. We hope that this will facilitate wider exploration of enantiopure sSPhos as a valuable chiral monodentate phosphine ligand. We anticipate that it will find use in industrial and academic laboratories, given the ubiquity of palladium catalysis in modern synthetic organic chemistry.

## Experimental Section

The following procedure details the protocol for the scaled-up synthesis of H*-(R)*-sSPhos and H*-(S)*-sSPhos, starting with SPhos (250 mmol) and using a Radleys reactor. Full details of optimization, reactions, and characterization can be found in the Supporting Information.

### Sulfonation to Obtain H*-(Rac)*-sSPhos

Trifluoroacetic anhydride (92 mL, 0.66 mol, 2.63 equiv) was added dropwise to sulfuric acid (18 mL, 0.34 mol, 1.25 equiv) using a dropping funnel (flow rate of 10 mL/min). Once complete, this biphasic mixture was stirred at room temperature for 17 h to form a homogeneous yellow solution of TFAA-H_2_SO_4_. *The homogeneous solution is typically formed after 4 h.* SPhos (102.65 g, 0.25 mol) was added portion-wise (10 g batches every minute) to a solution of TFAA-H_2_SO_4_ (95 mL, 0.27 mmol, 1.08 equiv) and TFA (150 mL) at a jacket temperature of 0 °C in a 2 L Radleys reactor to form a yellow solution, which was then stirred (200 rpm) at a jacket temperature of 20 °C for 17 h. The a*ddition of SPhos to this mixture resulted in a minor exotherm* (*∼5* ^*o*^ *C*). The reaction mixture was then concentrated under reduced pressure at a jacket temperature of 60 °C within the reactor to form a yellow gel. EtOAc (1 L) was added to form a white suspension, which was stirred (200 rpm) for 1 h at a jacket temperature of 20 °C. The suspension was removed from the reactor and filtered to yield a paste-like white solid. The solid was further dried under pressure to give H*-(rac)-*sSPhos as a free-flowing white powder (79.6 g, 0.16 mol, 65%).

### Salt Formation and First Recrystallization to Obtain Enriched QD*-(R)*-sSPhos and QD-*(S)*-sSPhos

H*-(rac)-*sSPhos (79.6 g, 0.16 mol), Quinidine (55.3 g, 0.17 mol, 1.05 equiv), and MeCN (2.90 L) were stirred (200 rpm) under reflux in a 5 L Radleys reactor at a jacket temperature of 100 °C (internal reaction temperature ∼83 °C). Once the milky-white suspension became a colorless/light yellow solution (25–40 min), the heating and stirring were stopped, and the reaction mixture was allowed to slowly cool for 17 h. *Note: The product recrystallizes on the vessel walls*. The filtrate was removed by decanting, to leave QD*-(R)-*sSPhos (51.4 g, 63.18 mmol, 39%, 96:04 d.r) remaining as a white powder stuck to the reactor. QD-*(R)-*sSPhos (96:04 d.r): ^**1**^ **H NMR** (700 MHz, CDCl_3_) δ 11.34 (s, 1H), 8.74 (d, *J* = 4.5 Hz, 1H), 8.00 (dd, *J* = 32.5, 9.0 Hz, 2H), 7.69 (d, *J* = 4.5 Hz, 1H), 7.53 (ddd, *J* = 7.7, 3.6, 1.5 Hz, 1H), 7.46–7.35 (m, 3H), 7.30 (dd, *J* = 9.2, 2.6 Hz, 1H), 7.28–7.22 (m, 1H), 6.68 (d, *J* = 8.9 Hz, 1H), 6.54 (d, *J* = 4.5 Hz, 1H), 6.04 (ddd, *J* = 17.4, 10.5, 7.3 Hz, 1H), 5.83 (br s, 1H), 5.28–5.19 (m, 2H), 4.25 (dd, *J* = 12.7, 8.3 Hz, 1H), 3.94 (s, 3H), 3.70 (s, 3H), 3.53 (t, *J* = 11.9 Hz, 1H), 3.49 (m, 1H), 3.39 (s, 3H), 3.36–3.32 (m, 2H), 2.59 (q, *J* = 8.8 Hz, 1H), 2.43 (m, 1H), 2.01–1.90 (m, 3H), 1.77–1.56 (m, 7H), 1.44 (m, 4H), 1.23–0.62 (m, 12H). ^**13**^ **C NMR** (176 MHz, CDCl_3_) δ 159.6, 158.9, 155.2, 146.8, 144.5, 144.0, 143.8, 141.5 (d, *J* = 27.9 Hz), 136.4, 132.4, 132.3 (m), 131.2, 131.0, 129.8 (m), 128.7, 126.9, 126.6, 125.9, 122.8, 118.8, 117.6, 105.0, 100.5, 66.4, 61.0, 60.2, 57.2, 55.5, 49.4, 48.5, 37.5, 35.4, 32.3, 29.6 (m), 29.0 (m), 27.6, 27.3 (m), 27.0 (d, *J* = 10.0 Hz), 26.7 (d, *J* = 12.0 Hz), 26.2, 25.9 (m), 23.4, 17.9. ^**31**^ **P NMR** (162 MHz, CDCl_3_) δ −8.73. *Cation exchange to H-sSPhos was performed before* **chiral SFC analysis** (IH-3, 70:30 CO_2_:MeOH, 2.5 mL/min, 2.21 min [major], 5.51 min [minor]).

The filtrate was concentrated under vacuum to obtain QD*-(S)-*sSPhos (78.77 g, 96.82 mmol, 61%, 18:82 d.r), as a light yellow solid. *QD-(S)-*sSPhos (18:82 d.r): ^**1**^ **H NMR** (700 MHz, CDCl_3_) δ 11.16 (s, 1H), 8.68 (dd, *J* = 4.5, 3.4 Hz, 1H), 8.11 (d, *J* = 8.7 Hz, 1H), 7.86 (dd, *J* = 9.1, 7.4 Hz, 1H), 7.65 (dd, *J* = 4.5, 0.7 Hz, 1H), 7.61–7.53 (m, 1H), 7.41 – 7.34 (m, 2H), 7.28–7.12 (m, 2H), 6.62 (d, *J* = 8.8 Hz, 1H), 6.40 (d, *J* = 3.1 Hz, 1H), 6.28 (s, 1H), 5.99 (ddd, *J* = 17.4, 10.4, 7.2 Hz, 1H), 5.28–5.05 (m, 1H), 4.23 (ddd, *J* = 13.2, 8.5, 2.4 Hz, 1H), 3.90 (d, *J* = 7.2 Hz, 3H), 3.62 (s, 2H), 3.46–3.42 (m, 3H), 3.39–3.30 (m, 2H), 3.23 (m, 1H), 3.11 (br s, 1H), 2.51 (m, 1H), 2.35 (ddt, *J* = 13.6, 9.8, 1.9 Hz, 1H), 2.06 (m, 2H), 1.94–1.82 (m, 2H), 1.80–1.36 (m, 13H), 1.33–0.84 (m, 12H). ^**13**^ **C NMR** (176 MHz, CDCl_3_) δ 163.0 (d, *J* = 35.0 Hz), 159.8, 159.7, 158.5, 155.6, 155.6, 155.5, 147.0, 144.5, 144.4, 143.8, 141.7, 141.7, 141.6 (d, *J* = 31.1 Hz), 135.4 (d, *J* = 15.3 Hz), 132.4 (d, *J* = 3.4 Hz), 132.0 (d, *J* = 6.4 Hz), 132.0, 131.3, 130.7, 130.6, 129.3, 129.3, 128.1, 128.1, 126.7, 126.5 (d, *J* = 6.3 Hz), 126.4, 125.6, 125.6, 122.4, 118.7, 117.6, 117.5, 116.4, 116.0, 105.0, 104.7, 100.2, 66.7, 66.5, 61.0, 61.0, 60.2, 60.1, 56.7, 56.7, 55.4, 50.3, 49.5, 49.4, 48.5, 37.4, 35.4 (d, *J* = 13.0 Hz), 35.2 (m), 32.7 (d, *J* = 10.8 Hz), 30.2 (d, *J* = 18.1 Hz), 29.6 (m), 29.5, 29.4 (m), 29.3 (m) 29.1 (d, *J* = 7.7 Hz), 27.7 (m), 27.6 (m), 27.5 (m), 27.54 (m), 27.1 (m), 27.1 (dd, *J* = 10.6, 6.1 Hz), 27.0, 26.4 (m) 26.3, 26.1, 25.1, 24.2, 23.9, 23.4, 18.0, 11.5, 1.9. ^**31**^ **P NMR** (162 MHz, CDCl_3_) δ −8.90. *Cation exchange to H-sSPhos was performed before* **chiral SFC analysis** (IH-3, 70:30 CO_2_:MeOH, 2.5 mL/min, 2.21 min [minor], 5.51 min [major]).

### Second Recrystallization to Obtain Highly Enriched QD*-(R)*-sSPhos

MeCN (2.90 L) was added back to the reactor containing the recrystallized QD*-(R)-*sSPhos (51.4 g, 63.18 mmol, 96:04 d.r) and again the solution was stirred under reflux at a jacket temperature of 100 °C (internal reaction temperature ∼83 °C). Once the milky-white suspension became a colorless/light yellow solution (25–40 min), the heating was stopped, and the reaction mixture was allowed to cool to r.t and stirred for 17 h. The filtrate was again decanted out of the reactor before more MeCN (50 mL) was added and decanted off, *leaving the product recrystallized as a white solid on the vessel walls*. The precipitated product was then extracted off the walls of the reactor by adding MeOH (200 mL) and refluxing at a jacket temperature of 80 °C while stirring (350 rpm) to form a solution of the product in MeOH. This solution was removed from the reactor and concentrated under vacuum, giving a light yellow/white solid, QD*-(R)-*sSPhos (41.12 g, 50.54 mmol, 80%, >99:1 d.r).

### Protonation to Obtain H*-(R)-*sSPhos

AmberLite IR120H (300 g) was washed with MeOH until the solution ran clear (red to colorless). Batches of QD*-(R)-*sSPhos (10 g, 12.21 mmol, > 99:1 d.r) were dissolved in MeOH (500 mL) to give a light yellow/colorless solution and flushed through the washed AmberLite 12 times. Once complete, the solution was concentrated under vacuum to yield H*-(R)-*sSPhos (5.7 g, 11.6 mmol, 95%, >99% ee) as a pale light yellow solid. Chiral SFC Analysis (IH-3, 70:30 CO2:MeOH, 2.5 mL/min, 2.21 min [major], 5.51 min [minor])^.**1**^ **H NMR** (700 MHz, MeOD) δ 8.07 (d, *J* = 8.9 Hz, 1H), 7.99 (dd, *J* = 11.0, 8.1 Hz, 1H), 7.91 (t, *J* = 7.2 Hz, 1H), 7.76 (t, *J* = 6.7 Hz, 1H), 7.69 (d, *J* = 4.6 Hz, 1H), 7.05 (d, *J* = 8.9 Hz, 1H), 3.82 (s, 1H), 3.40 (s, 3H), 3.02 (q, *J* = 11.6 Hz, 3H), 2.52 (q, *J* = 12.2 Hz, 1H), 2.08 (m, 1H), 2.00–1.92 (m, 1H), 1.92–1.86 (m, 2H), 1.79 (m, 3H), 1.72–1.59 (m, 4H), 1.57–1.24 (m, 6H), 1.08 (m, 3H). ^**13**^ **C NMR** (176 MHz, MeOD) δ 159.6 (d, *J* = 0.8 Hz), 154.0, 140.2 (d, *J* = 5.7 Hz), 134.4 (d, *J* = 9.0 Hz), 133.9 (d, *J* = 3.1 Hz), 132.9 (d, *J* = 10.1 Hz), 131.7, 131.1, 128.6 (d, *J* = 12.0 Hz), 121.6 (d, *J* = 4.5 Hz), 113.4 (d, *J* = 80.0 Hz), 106.7, 28.8 (d, *J* = 43.6 Hz), 27.2 (d, *J* = 43.5 Hz), 26.5 (m), 26.4 (d, *J* = 2.2 Hz), 25.7 (d, *J* = 6.7 Hz), 25.6 (d, *J* = 6.1 Hz), 25.4 (d, *J* = 14.0 Hz), 25.1 (d, *J* = 13.7 Hz), 24.8 (d, *J* = 2.1 Hz), 24.8 (d, *J* = 2.2 Hz), 24.7 (d, *J* = 4.1 Hz). ^**31**^ **P NMR** (162 MHz, MeOD) δ 17.3 (br t). **Chiral SFC Analysis** (IH-3, 70:30 CO_2_:MeOH, 2.5 mL/min, 2.21 min [major], 5.51 min [minor]). $\left[\alpha \right]\frac{25}{D}=-107.4\left(c0.12,\mathrm{MeOH}\right)$ .

### Protonation to Obtain H*-(S)-*sSPhos

AmberLite IR120H (150 g) was washed with MeOH until the solution ran clear (red to colorless). Batches of QD*-(S)-*sSPhos (10 g, 12.21 mmol, 18:82 d.r) were dissolved in MeOH (500 mL) and flushed through the washed AmberLite 8 times. Once complete, the solution was concentrated under vacuum. MeCN (500 mL) was added and sonicated before being concentrated under vacuum; this was repeated twice. MeCN (500 mL) was again added and sonicated to give a a milky-white suspension, which was filtered to give enantioenriched H*-(S)-*sSPhos (2.4 g, 4.9 mmol, 40%,1:99 e.r) as a pasty white solid, which was dried under vacuum to give a free-flowing solid. **Chiral SFC Analysis** (IH-3, 70:30 CO_2_:MeOH, 2.5 mL/min, 2.21 min [minor], 5.51 min [major]). $\left[\alpha \right]\frac{25}{D}=+104.9\left(c0.12,\mathrm{MeOH}\right)$ .

## Supplementary Material



## Notes

Experimental details for various reaction scales; spectroscopic data of novel isolated compounds and key synthetic intermediates; and tables of optimization screens of reaction conditions and XRPD analysis (PDF)
