# Supplementary material for: Optimization of a Resolution Process Allowing Access to Both Enantiomers of the Versatile Chiral Phosphine Ligand sSPhos on Scale
Source: Org Process Res Dev. 2026 Jun 25;30(7):1987–95. doi: 10.1021/acs.oprd.6c00142 (PMC13386642; doi:10.1021/acs.oprd.6c00142)

Optimization of a Resolution Process Allowing Access to Both Enantiomers of the Versatile Chiral Phosphine Ligand sSPhos on Scale

Hamzah Sharif,[a] Thomas D. Svejstrup,[b] Staffan Karlsson,[b] and Robert J. Phipps [a*]

[a] Yusuf Hamied Department of Chemistry, University of Cambridge, Lensfield Road, Cambridge, CB2 1EW, UK.

[b] Early Chemical Development, Pharmaceutical Sciences, R&D, AstraZeneca, Gothenburg 431 83, Sweden

Table of Contents

General Information 3

Optimization of Sulfonation of SPhos using H_2SO_4 4

Optimization of Diastereoselective Recrystallization of QD-*(rac)*-sSPhos 5

Solubility Comparison 10

Attempted enantio-enrichment of H-sSPhos (18:82) by washing with MeCN 10

Full Cation Exchange of QD-sSPhos to H-sSPhos 11

Full Procedure: 10 mmol scale using Laboratory Equipment 11

Full Procedure: 50 mmol scale using a Radleys® Reactor 12

Full Procedure: 250 mmol scale using a Radleys® Reactor 13

sSPhos Characterization Data: 14

Enantioselective Suzuki-Miyaura Coupling... 16

General Information

Reagents: All reagents, unless otherwise stated, were used as supplied from commercial sources without further purification. MeCN and toluene were distilled from calcium hydride. H_2SO_4 99.999% from Sigma Aldrich (product code: 339741) was used for all sulfonation reactions. H_2SO_4 >=95% (produce code: 10294300) from Fisher Chemicals performed the same as the purer grade. AmberLite IR120H from Sigma Aldrich (product code: 06428) was used for all cation exchanges and weighed as the wet resin.

Reaction setup: 0.1 and 1 mmol reactions were carried out in 4 mL, 15x45mm and 20 mL, 23x75.5mm crimp-top vials, respectively. In cases where the reactions were heated, the vials were heated in deep welled heating blocks. For the 10 mmol scale up reaction, standard glass round bottom flasks were used. In cases where the reaction was heated, an air-open reflux condenser and heating block was used. For the 50 and 250 mmol scale up reactions, 1,2,3 or 5 L Radleys® reactors were used under nitrogen. In cases where the reaction was heated, an jacket temperature was set.

NMR spectra: ^{1}H NMR spectra were recorded on a 700 MHz TXO Cryoprobe, 600 MHz Bruker Avance DRX-600 spectrometer, 500 MHz Bruker DCH Cryoprobe, 400 MHz Bruker QNP Cryoprobe or 400 MHz Bruker Avance NEO Prodigy N_2 Cryoprobe. Chemical shifts are reported in parts per million (ppm) and the spectra are calibrated to the resonance resulting from incomplete deuteration of the solvent ($CDCl_3$: 7.26 ppm; DMSO-d^6: 2.50 ppm, qn; MeOD-d^4: 3.31 ppm, qn). ^{13}C NMR spectra were recorded on the same spectrometers with complete proton decoupling. Chemical shifts are reported in ppm with the solvent resonance as the internal standard ($CDCl_3$: 77.16 ppm, t; DMSO-d^6: 39.52 ppm, sept; MeOD-d^4: 49.00 ppm, sept). Data are reported as follows: chemical shift δ/ppm, multiplicity (s = singlet, d = doublet, t = triplet, q = quartet, qn = quintet, sept = septet, br = broad, m = multiplet or combinations thereof; ^{13}C, ^{19}F and ^{31}P signals are singlets unless otherwise stated), coupling constants J in Hz, integration (1H only). ^{1}H-COSY, DEPT-135, HMQC and HMBC were used where appropriate to facilitate structural determination. The carbon atom attached to boron was generally not observed by ^{13}C spectroscopy due to quadrupolar relaxation. ^{19}F and ^{31}P NMR spectra were recorded on a 400 MHz Bruker Avance III HD and 400 MHz Bruker Avance NEO Prodigy N_2 Cryoprobe Spectrometer with complete proton decoupling.

LCMS analysis: Performed on Waters Acquity UPLC system equipped with Waters PDA, sample manager, sample organizer, column oven and Waters Xevo QTOF mass spectrometer. A 3 min method was run with acetonitrile and pH 3 formic acid buffer, at a 2 mL/min flowrate, at a 90:10 to 0:100 (buffer:acetonitrile) gradient. sSPhos had a retention time of 1.01 min and SPhos 1.61 min.

Chiral SFC analysis: Performed on a Waters ACQUITY UPC2 System with a DAICEL CHIRALPAK IH column (4.6 × 250 mm, 3.0 μm) in a mixed solvent system of supercritical CO_2 and MeOH, A system backpressure of 138 bar was used.

Chiral HPLC analysis: Performed on a Waters ARC system with a CHIRALART SC column (4.6 x 250 mm, 3 μm) in a mixed solvent system of n-hexane and iPrOH.

SFC analysis of Diastereomers: Diastereomeric ratios (d.r) were assigned from the enantiomer ratios (e.r) of H-sSPhos, after removing the chiral cations using AmberLite IR120H.

Optimization of Sulfonation of SPhos using H_2SO_4

SPhos (410 mg, 1 mmol) was added portion wise to the sulfonating agent at 0 °C. Once complete the reaction was allowed to react at the specified temperature and time. The yield of product was determined by LCMS analysis (retention time 1.01, *see general information for more details*) using the percentage integration of the peak areas. EtOAc (5 mL) was added and stirred for 5 minutes then if a solid was produced, this was filtered to give the product. A sticky white solid was usually obtained. *Note: reproducibility of this method remained low, with a yellow gel sometimes being formed, instead of a white solid.*

Table S1.

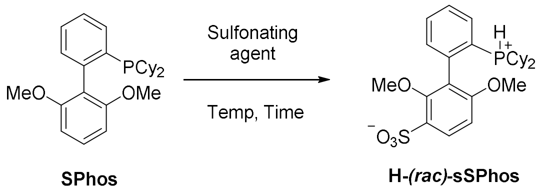

Entry	Sulfonating agent	Temp	X h	LCMS Yield	Isolated Yield
Lit.	H_2SO_4 (0.065 ml, 1.2 mmol, 1.2 eq.) in DCM (0.4 mL)	40 °C	17	63%	-
1	H_2SO_4 (1.41 ml, 26 mmol, 26 eq.)	40 °C	17	Quant	>100%
2	H_2SO_4 (1.41 ml, 26 mmol, 26 eq.)	r.t	17	Quant	>100%
3	H_2SO_4 (1.41 ml, 26 mmol, 26 eq.)	r.t	1	Quant	>100%
4	H_2SO_4 (0.71 ml, 13 mmol, 13 eq.)	r.t	17	Quant	>100%
5	H_2SO_4 (0.16 ml, 3 mmol, 3 eq.)	r.t	17	43%	-

Optimization of Sulfonation of SPhos using Activated H_2SO_4

Acid anhydride (2.24 mmol, 2.24 eq.), was added dropwise to sulfuric acid (0.065 ml, 1.12 mmol) and was stirred at r.t for 17 h. SPhos (410 mg, 1 mmol) was added portion wise to the activated sulfonating agents (1.12 mmol) in solvent (0.6 mL) at 0 °C, then stirred at r.t for the specified time.. The yield of product was determined by LCMS analysis (retention time 1.01, *see general information for more details*) using the percentage integration of the peak areas. Once complete, the solution was concentrated under vacuum to produce a yellow gel, EtOAc (5 mL) was added and stirred for 5 minutes then, if a solid was produced, this was filtered to give the product.

Table S2.

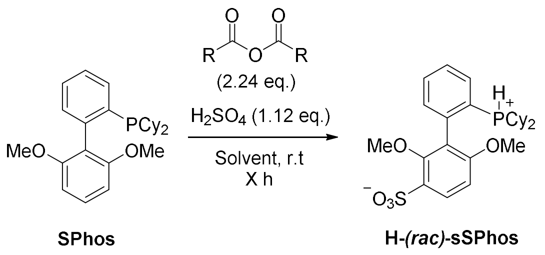

Entry	R	Acid Anhydride	Solvent	X h	LCMS Yield	Isolated Yield
1	Me	Acetic anhydride (0.21 mL)	TFA	17	0%	-
2	^{t}Bu	Pivolic anhydride (0.46 mL)	TFA	17	0%	-
3	Ph	Benzoic anhydride (0.43 mL)	TFA	17	0%	-

4	CF_3	TFAA (0.31 mL)	TFA	17	quant	95%
5	CF_3	TFAA (0.31 mL)	Acetic acid	17	67%	-
6	CF_3	TFAA (0.31 mL)	EtOAc	40	67%	-

Optimization of Diastereoselective Recrystallization of QD-*(rac)*-sSPhos

Solvent Evaluation:

H-*(rac)*-sSPhos (49.0 mg, 0.1 mmol), quinidine (34 mg, 0.105 mmol, 1.05 eq.) and solvent (1.75 mL) were stirred at a heating block temperature of 110 °C. Once/if the suspension became a colourless/light yellow solution, the heating was stopped and the reaction mixture was allowed to cool to r.t and stirred for 17 h. Any solid that precipitated out was filtered and converted to H-sSPhos (see method below), before being analysed by chiral SFC. **Chiral SFC Analysis** (IH-3, 70:30 CO_2:MeOH, 2.5 mL/min, 2.21 min [major], 5.51 min [minor]). *The d.r of QD-sSPhos was inferred from the e.r of H-sSPhos.*

AmberLite IR120H (1.5 g) was washed with MeOH till the solution ran clear. QD-*(R)*-sSPhos (precipitated solid) was dissolved in MeOH (2 mL) and flushed through the washed AmberLite 12 times. Once complete the solution was concentrated under vacuum to yield H-*(R)*-sSPhos.

Table S3.

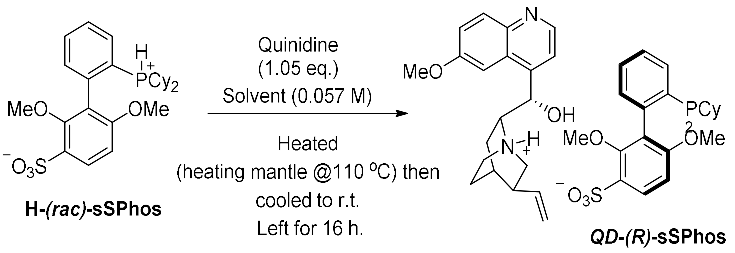

Entry	Solvent	Soluble at 110°C?	Yield	d.r
1	MeCN	Yes	42%	96:04
2	Me-THF	No	-	-
3	EtOAc	No	-	-
4	Acetone	No	-	-
5	EtOH	Yes	<5%	-

Concentration Evaluation:

H-*(rac)*-sSPhos (49.0 mg, 0.1 mmol), quinidine (34 mg, 0.105 mmol, 1.05 eq.) and MeCN were stirred at a heating block temperature of 110 °C. Once the suspension became a colourless/light yellow solution, the heating was stopped and the reaction mixture was allowed to cool to r.t and stirred for 17 h. Solid that precipitated out was filtered and converted to H-sSPhos (see method below), before being analysed by chiral SFC. **Chiral SFC Analysis** (IH-3, 70:30 CO_2:MeOH, 2.5 mL/min, 2.21 min [major], 5.51 min [minor]). *The d.r. of QD-sSPhos was inferred from the measured e.r. of H-sSPhos.*

AmberLite IR120H (1.5 g) was washed with MeOH till the solution ran clear. QD-*(R)*-sSPhos (precipitated solid) was dissolved in MeOH (2 mL) and flushed through the washed AmberLite 12 times. Once complete the solution was concentrated under vacuum to yield H-*(R)*-sSPhos.

Table S4.

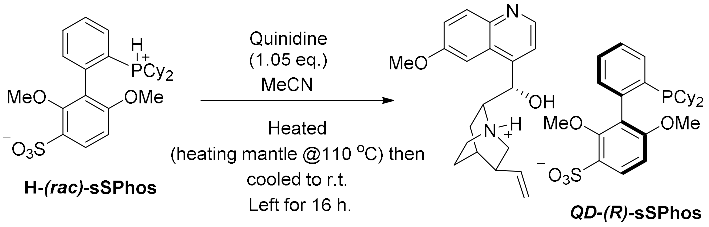

Entry	MeCN	Soluble at 110°C?	Yield	d.r
1	1.43 mL, 0.07 M	Yes	42%	85:15
2	1.75 mL, 0.057 M	Yes	42%	96:04
3	2.00 mL, 0.05 M	Yes	34%	96:04

Temperature Screen:

H-*(rac)*-sSPhos (49.0 mg, 0.1 mmol), quinidine (34 mg, 0.105 mmol, 1.05 eq.) and MeCN were stirred at a heating block temperature of 110 °C. Once the suspension became a colourless/light yellow solution, the heating was set to the mentioned End temperature and the reaction mixture was allowed to reach that temperature and then stirred for the mentioned time. Any solid that precipitated out was filtered and converted to H-sSPhos (see method below), before being analysed by chiral SFC. **Chiral SFC Analysis** (IH-3, 70:30 CO_2:MeOH, 2.5 mL/min, 2.21 min [major], 5.51 min [minor]). *The d.r. of QD-sSPhos was inferred from the measured e.r. of H-sSPhos.*

AmberLite IR120H (1.5 g) was washed with MeOH till the solution ran clear. QD-*(R)*-sSPhos (precipitated solid) was dissolved in MeOH (2 mL) and flushed through the washed AmberLite 12 times. Once complete the solution was concentrated under vacuum to yield H-*(R)*-sSPhos.

Table S5.

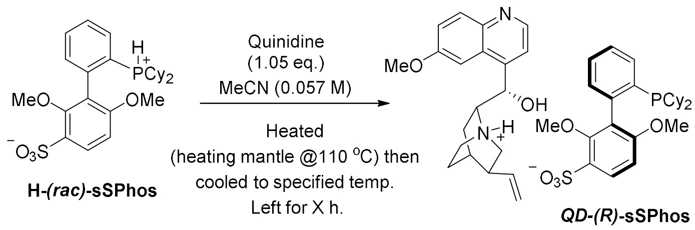

Entry	End T °C	X h	Yield	d.r
1	r.t	17	42%	96:04
2	40	17	10%	97:03
3	40	30	20%	92:08

Temperature-Solubility Curve:

QD-*(R)*-sSPhos/ QD-*(S)*-sSPhos (49.0 mg, 0.1 mmol) was dissolved in MeCN (1.75 mL) and stirred at various heating block temperatures, for 15 min, before being filtered. A portion (0.1 mL) of this solution was diluted in MeCN (9.9 mL) to achieve 100-fold dilution. The sample was then run on the LCMS. The measured

integration value was compared against the integration of a 100-fold diluted standard sample of original concentration 28 mg/ml (as shown below). The concentration (mg/ml) was then able to be calculated.

For QD-*(R)*-sSPhos and QD-*(S)*-sSPhos a standard sample of 28 mg/ml concentration (49.0 mg, 0.1 mmol in 1.75 ml MeCN) was prepared and then diluted 100-fold to be run on the LCMS with integrations recorded (shown below).

	concentration	LCMS integration 1	LCMS integration 2	LCMS integration 3	LCMS integration average/ standard value
QD-*(R)*-sSPhos	0.28 mg/mL	188154	187992	187890	188012
QD-*(S)*-sSPhos	0.28 mg/mL	187800	187972	188014	187928

%solubility = (LCMS integration/LCMS integration of standard)*100

%solubility QD-*(R)*-sSPhos = (LCMS integration/188012)*100

%solubility QD-*(S)*-sSPhos = (LCMS integration/187928)*100

Concentration (mg/ml) QD-*(R)*-sSPhos = (%solubility QD-*(R)*-sSPhos) *28/100

Concentration (mg/ml) QD-*(S)*-sSPhos = (%solubility QD-*(S)*-sSPhos) *28/100

Table S6.

QD-*(R)*-sSPhos				QD-*(S)*-sSPhos			
Heating Block Temperature	% solubility	Concentration (mg/ml)	LCMS Integration	Heating Block Temperature	% solubility	Concentration (mg/ml)	LCMS Integration
r.t	6	1.7	10693	r.t	34	9.5	63914
30 °C	4	1.1	8018	30 °C	36	10.1	67575
40 °C	4	1.1	7974	40 °C	38	10.6	71711
50 °C	4	1.1	7837	50 °C	36	10.1	67302
60 °C	4	1.1	6960	60 °C	34	9.5	63992
70 °C	5	1.4	9797	70 °C	34	9.5	63304
80 °C	8	2.2	14862	80 °C	38	10.6	70326
90 °C	21	5.9	38948	90 °C	55	15.4	103000
100 °C	60	16.8	113379	100 °C	100	28	187860
110 °C	100	28	188410	110 °C	100	28	188160

Chiral Base Screen:

H-*(rac)*-sSPhos (49.0 mg, 0.1 mmol), chiral amine (0.105 mmol, 1.05 eq.) and MeCN (1.75 mL) were stirred at a heating block temperature of 110 °C. Once/if the suspension became a colourless/light yellow solution, the heating was stopped and the reaction mixture was allowed to cool to r.t and stirred for 17 h. Any solid that precipitated out was filtered and converted to H-sSPhos (see method below), before being analysed by chiral SFC. **Chiral SFC Analysis** (IH-3, 70:30 CO_2:MeOH, 2.5 mL/min, 2.21 min [major], 5.51 min [minor]). *The d.r. of QD-sSPhos was inferred from the measured e.r. of H-sSPhos.*

AmberLite IR120H (1.5 g) was washed with MeOH till the solution ran clear. The precipitated solid was dissolved in MeOH (2 mL) and flushed through the washed AmberLite 12 times. Once complete the solution was concentrated under vacuum to yield H-sSPhos.

Table S7.

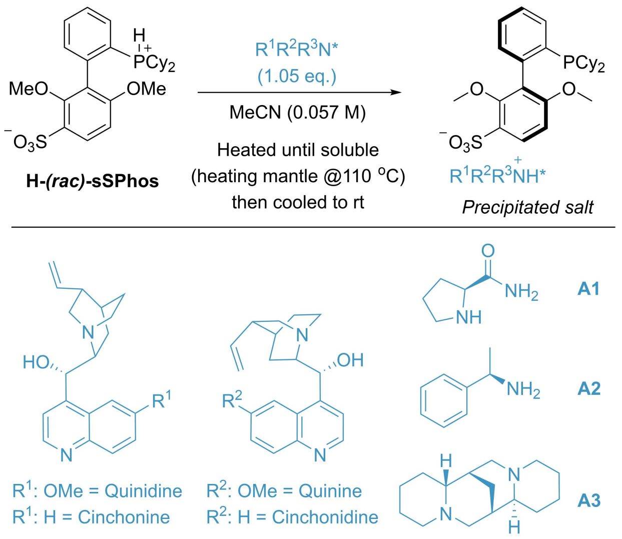

Entry	Chiral Amine	Soluble at 110°C?	Yield	d.r
1	QD (34.0 mg)	Yes	42%	96:04
2	QN (34.0 mg)	Yes	< 5%	-
3	CD (31.0 mg)	Yes	< 5%	-
4	CN (31.0 mg)	Yes	12%	*rac*
5	A1 (12.0 mg)	No	-	-
6	A2 (12.7 mg)	Yes	< 5%	-
7	A3 (24.6 mg)	Yes	< 5%	-

Optimization of Enantioenrichment of H-*(S)*-sSPhos through Partial Cation Removal

AmberLite IR120H (15g per 1g of QD-*(S)*-sSPhos) was washed with MeOH till the washings ran clear. QD-*(S)*-sSPhos (18:82, d.r.) was dissolved in MeOH (50 mL per 1g of QD-*(S)*-sSPhos) and flushed through the washed AmberLite 8 times. Once complete the solution was concentrated under vacuum. Filtration Solvent (50 mL per 1g of QD-*(S)*-sSPhos) was added and sonicated before being concentrated under vacuum, this was repeated twice. Filtration Solvent (50 mL per 1g of QD-*(S)*-sSPhos) was again added and sonicated to give a solid precipitation which was filtered to give enantioenriched H-*(S)*-sSPhos as a white solid. **Chiral SFC Analysis** (IH-3, 70:30 CO_2:MeOH, 2.5 mL/min, 2.21 min [minor], 5.51 min [major]).

Table S8.

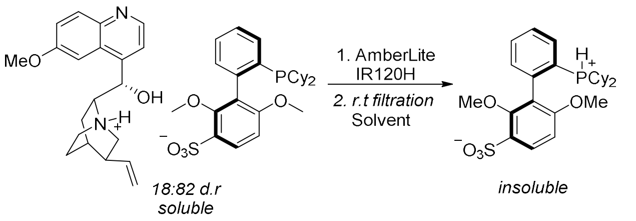

Scale	Filtration Solvent	Yield	e.r
2g, 2.45 mmol	EtOAc (100 mL x3)	40%	09:91
2g, 2.45 mmol	MeCN (100 mL x3)	38%	01:99
10g, 12.25 mmol	MeCN (500 mL x3)	40%	01:99

Investigation into other proton sources for protonation, besides AmberLite IR120H was performed:

QD-*(S)*-sSPhos (1g, 1.23 mmol, 18:82, e.r) and the specified acid were dissolved in MeOH (50 mL) and stirred at r.t for the mentioned time. After the specified time the solution was concentrated under vacuum. MeCN (50 mL) was added and the solution sonicated before being concentrated under vacuum, this was repeated twice. MeCN (50 mL) was again added and sonicated to give a solid precipitation which was filtered to give enantioenriched H-*(S)*-sSPhos as a white solid. **Chiral SFC Analysis** (IH-3, 70:30 CO_2:MeOH, 2.5 mL/min, 2.21 min [minor], 5.51 min [major]).

Table S9.

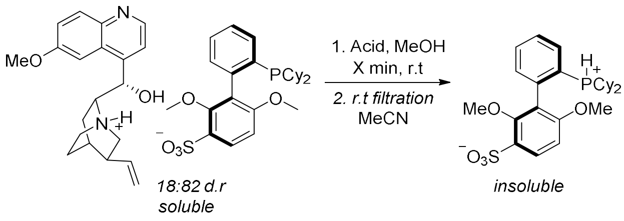

Entry	Acid	Comments	X min	Yield	%ee
1	HCl in dioxane (0.184 mL, 4 M, 0.736 mmol, 0.6 eq.)		15 min	240 mg, 0.49 mmol, 40%	98% ee
2	TFA (0.056 mL, 0.736 mmol, 0.6 eq.)		15 min	220 mg, 0.45 mmol, 37%	98% ee

3	Tosic acid.H_2O (140 mg, 0.736 mmol, 0.6 eq.)		15 min	270 mg, 0.55 mmol, 45%	98% ee
4	Tosic acid.H_2O (280 mg, 1.46 mmol, 1.2 eq.)		15 min	340 mg, 0.69 mmol, 56%	98% ee
5	Tosic acid.H_2O (560 mg, 2.92 mmol, 2.4 eq.)		15 min	375 mg, 0.77 mmol, 62%	98% ee
6	Tosic acid.H_2O (560 mg, 2.92 mmol, 2.4 eq.)		60 min	380 mg, 0.78 mmol, 63%	98% ee
7	Tosic acid.H_2O (2.33 g, 12.3 mmol, 10 eq.)		15 min	0%. Yellow oil obtained, with no precipitation after addition of MeCN	-
8	Tosic acid.H_2O (560 mg, 2.92 mmol, 2.4 eq.)	No sonication	15 min	362 mg, 0.75 mmol, 60%	98% ee

Solubility Comparison

Table S10.

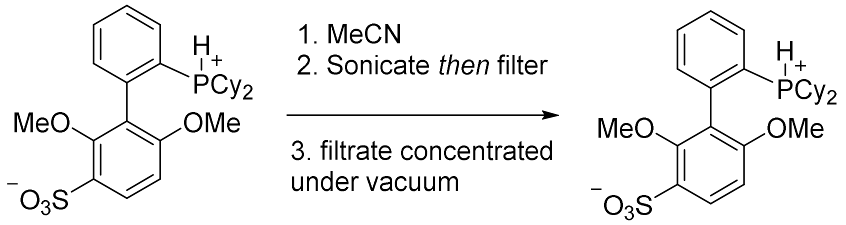

H-sSPhos	Mass of H-sSPhos dissolved in filtrate	% solubility	Concentration
H-*(rac)*-sSPhos (>99% ee)	2.0 mg	4%	0.4 mg/ml
H-*(R)*-sSPhos (>99% ee)	0.3 mg	0.6%	0.06 mg/ml
H-*(S)*-sSPhos (>99% ee)	0.4 mg	0.8%	0.08 mg/ml

MeCN (5 mL) was added to H-sSPhos (49 mg, 0.1 mmol) at r.t and sonicated for 1 minute. A milky-white emulsion formed which was filtered, collecting and concentrating the filtrate under vacuum. The mass of H-sSPhos dissolved in the filtrate was measured and used to determine the solubility of the different enantiomers and racemate.

Attempted enantio-enrichment of H-sSPhos (18:82) by washing with MeCN

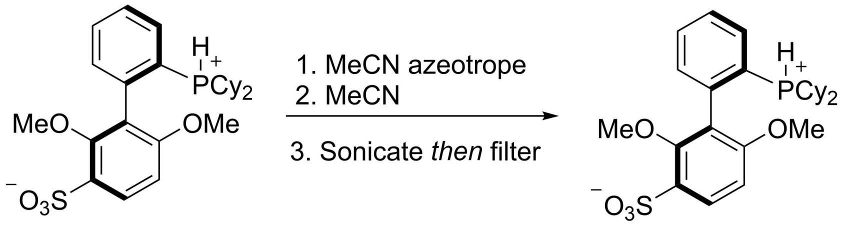

64% ee

98%, 64% ee

AmberLite IR120H (60g) was washed with MeOH till the solution ran clear. QD-*(S)*-sSPhos (2g) was dissolved in MeOH (100 mL) and flushed through the washed AmberLite 12 times. Once complete the solution was concentrated under vacuum to yield H-*(S)*-sSPhos (570 mg, 1.17 mmol , 95%, 64% ee) as a pale light yellow solid. **Chiral SFC Analysis** (IH-3, 70:30 CO_2:MeOH, 2.5 mL/min, 2.21 min [minor], 5.51 min [major]). MeCN (100 mL) was added and the solution sonicated before being concentrated under vacuum, this was repeated twice. MeCN (100 mL) was again added and sonicated to give a solid precipitation which

was filtered to give H-*(S)*-sSPhos (559 mg, 1.15 mmol , 98%, 64% ee). **Chiral SFC Analysis** (IH-3, 70:30 CO_2:MeOH, 2.5 mL/min, 2.21 min [minor], 5.51 min [major]).

Full Cation Exchange of QD-sSPhos to H-sSPhos

AmberLite IR120H (30g per 1g of QD-sSPhos) was washed with MeOH till the solution ran clear. QD-sSPhos was dissolved in MeOH (50 mL per 1g of QD-sSPhos) and flushed through the washed AmberLite 12 times. Once complete the solution was concentrated under vacuum to yield H-sSPhos (>99% ee) as a pale light yellow solid. **Chiral SFC Analysis** (IH-3, 70:30 CO_2:MeOH, 2.5 mL/min, 2.21 min [major], 5.51 min [minor]).

Full Procedure: 10 mmol scale using Laboratory Equipment

QD-*(R/S)*-sSPhos:

Trifluoroacetic anhydride (3.68 ml, 26.3 mmol, 2.63 eq.) was added dropwise to sulfuric acid (0.72 ml, 12.5 mmol, 1.25 eq.), using a dropping funnel (flow rate of 10 mL/min). Once complete this biphasic mixture was stirred at room temperature for 17 h to form a homogenous yellow solution of TFAA-H_2SO_4. *The homogenous solution is typically formed after 4 hours.* SPhos (4.10 g, 10 mmol) was added portion wise to a solution of TFAA-H_2SO_4 (3.8 mL, 10.8 mmol, 1.08 eq.) and TFA (6 mL) at 0 °C, then stirred at r.t for the 17 h. The reaction mixture was then concentrated under reduced pressure to form a yellow gel. EtOAc (40 ml) was added to form a white suspension, which was stirred for 10 minutes. The suspension was then filtered and the solid further concentrated under pressure, to give a H-*(rac)*-sSPhos as a white powder (4.66 g, 9.5 mmol, 95%). H-*(rac)*-sSPhos (4.66 g, 9.5 mmol), Quinidine (3.2 g, 9.98 mmol, 1.05 eq.) and MeCN (158 ml) were stirred under reflux, at a heating block temperature of 110 °C. Once the suspension became a colourless/light yellow solution, the heating was stopped and the reaction mixture was allowed to cool to r.t and stirred for 17 h. *Note: The product recrystalizes to the vessel walls*. The filtrate was removed by decanting to leave QD-*(R)*-sSPhos (3.25 g, 3.99 mmol, 42%, 96:04 d.r) remaining in the flask. A small portion of the precipitate was taken and converted to H-sSPhos (method below) and analysed by chiral SFC. **Chiral SFC Analysis** (IH-3, 70:30 CO_2:MeOH, 2.5 mL/min, 2.21 min [major], 5.51 min [minor]). The filtrate was concentrated under vacuum to obtain QD-*(S)*-sSPhos (4.65g, 5.51 mmol, 58%, 15:85 d.r). Again a small portion was taken and converted to H-sSPhos (method below) and analysed by chiral SFC. **Chiral SFC Analysis** (IH-3, 70:30 CO_2:MeOH, 2.5 mL/min, 2.21 min [minor], 5.51 min [major]). *The d.r. of QD-sSPhos was inferred from the measured e.r. of H-sSPhos.*

AmberLite IR120H (1.5 g) was washed with MeOH till the solution ran clear. QD-sSPhos (50 mg, 0.06 mmol) was dissolved in MeOH (1 mL) and flushed through the washed AmberLite 12 times. Once complete the solution was concentrated under vacuum to yield H-sSPhos.

H-*(R)*-sSPhos:

MeCN (158 ml) was added back to the vessel containing the recrystallized QD-*(R)*-sSPhos (3.25 g, 3.99 mmol, 96:04 d.r) and again the solution was stirred under reflux, at a heating block temperature of 110 °C. Once the suspension became a colourless/light yellow solution, the heating and stirring was stopped and the reaction mixturewas allowed to slowly cool for 17 h. The precipitated product was then scratched off the walls of the vessel, to form a suspension, which was filtered, collecting the white solid, QD-*(R)*-sSPhos (2.6 g, 3.19 mmol, 80%, >99:1 d.r). AmberLite IR120H (78 g) was washed with MeOH till the solution ran clear. QD-*(R)*-sSPhos (2.6 g, 3.19 mmol, >99:1 d.r) was dissolved in MeOH (130 mL) and flushed through the washed AmberLite 12 times. Once complete the solution was concentrated under vacuum to yield H-*(R)*-sSPhos (1.5 g, 3.03 mmol, 95%, >99% ee) as a pale light yellow solid. **Chiral SFC Analysis** (IH-3, 70:30 CO_2:MeOH, 2.5 mL/min, 2.21 min [major], 5.51 min [minor]).

H-*(S)*-sSPhos:

AmberLite IR120H (70 g) was washed with MeOH till the solution ran clear. QD-*(S)*-sSPhos (4.65g, 5.51 mmol, 15:85 d.r) was dissolved in MeOH (232 mL) and flushed through the washed AmberLite 8 times. Once complete the solution was concentrated under vacuum. MeCN (233 ml) was added and sonicated before being concentrated under vacuum, this was repeated twice. MeCN (233 ml) was again added and sonicated to give a solid precipitation, which was filtered to give enantioenriched H-*(S)*-sSPhos (1.1 g, 2.1 mmol, 39%,1:99 e.r) as a white solid. **Chiral SFC Analysis** (IH-3, 70:30 CO_2:MeOH, 2.5 mL/min, 2.21 min [minor], 5.51 min [major]).

Full Procedure: 50 mmol scale using a Radleys® Reactor

QD-*(R/S)*-sSPhos:

Trifluoroacetic anhydride (18.4 ml, 131.5 mmol, 2.63 eq.) was added dropwise to sulfuric acid (3.6 ml, 62.5 mmol, 1.25), using a dropping funnel (flow rate of 10 mL/min). Once complete this biphasic mixture was stirred at room temperature for 17 h to form a homogenous yellow solution of TFAA-H_2SO_4. *The homogenous solution is typically formed after 4 hours.* SPhos (20.5 g, 50 mmol) was added portion wise to a solution of TFAA-H_2SO_4 (19 mL, 54 mmol, 1.08 eq.) and TFA (30 mL) at a jacket temperature of 0 °C in a 1L Radleys® reactor, to form a yellow solution, which was then stirred (100 rpm) at a jacket temperature of 20 °C for the 17 h. *Addition of SPhos to this mixture resulted in a minor exotherm, ~5 °C.* The reaction mixture was then concentrated under reduced pressure at a jacket temperature of 60 °C, within the reactor to form a yellow gel. EtOAc (200 ml) was added to form a milky-white suspension, which was stirred (100 rpm) at a jacket temperature of 20 °C, for 30 minutes. The suspension was then removed from the reactor and filtered to yield a paste-like white solid. The solid was further dried under pressure, to give a H-*(rac)*-sSPhos as a free-flowing white powder (24.0 g, 49 mmol, 98%). H-*(rac)*-sSPhos (24.0 g, 49 mmol), Quinidine (16.7 g, 51.45 mmol, 1.05 eq.) and MeCN (813 ml) were stirred (100 rpm) under reflux in a 2L Radleys® reactor, at jacket temperature of 100 °C (Internal reaction temperature ~83 °C). Once the milky-white suspension became a colourless/light yellow solution, the heating was stopped and the reaction mixture was allowed to cool to r.t and stirred for 17 h. *Note: The product recrystalizes to the vessel walls*. The filtrate was removed by decanting to leave QD-*(R)*-sSPhos (17.2 g, 21.1 mmol, 43%, 96:04 d.r) as a white solid remaining in the reactor. A small portion of the precipitate was taken and converted to H-sSPhos (method below) and analysed by chiral SFC. **Chiral SFC Analysis** (IH-3, 70:30 CO_2:MeOH, 2.5 mL/min, 2.21 min [major], 5.51 min [minor]). The filtrate was concentrated under vacuum to obtain QD-*(S)*-sSPhos (22.7 g, 27.9 mmol, 57%, 15:85 d.r), as a light yellow solid. Again a small portion was taken and converted to H-sSPhos (method below) and analysed by chiral SFC. **Chiral SFC Analysis** (IH-3, 70:30 CO_2:MeOH, 2.5 mL/min, 2.21 min [minor], 5.51 min [major]). *The d.r. of QD-sSPhos was inferred from the measured e.r. of H-sSPhos.*

AmberLite IR120H (1.5 g) was washed with MeOH till the solution ran clear. QD-sSPhos (50 mg, 0.06mmol) was dissolved in MeOH (1 mL) and flushed through the washed AmberLite 12 times. Once complete the solution was concentrated under vacuum to yield H-sSPhos.

H-*(R)*-sSPhos:

MeCN (813 ml) was added back to the reactor containing the recrystallized QD-*(R)*-sSPhos (17.2 g, 21.1 mmol, 96:04 d.r) and again the solution was stirred (100 rpm) under reflux, at a jacket temperature of 100 °C (Internal reaction temperature ~83 °C). Once the milky-white suspension became a colourless/light yellow solution (25-40 min), the heating was stopped and the reaction mixture was allowed to cool to r.t and stirred for 17 h. The filtrate was again decanted out of the reactor, before more MeCN (50 ml) was added

and decanted off. *Leaving the product recrystalized as a white-solid on the vessel walls*. The precipitated product was then extracted off the walls of the reactor, by adding MeOH (100 mL) and refluxing at a jacket temperature of 80 °C and stirring (250 rpm) to form a pale light yellow solution of the product in MeOH. This solution was removed from the reactor and concentrated under vacuum giving a pale light yellow/ white solid, QD-*(R)*-sSPhos (14.0 g, 17.1 mmol, 81%, >99:1 d.r). AmberLite IR120H (300 g) was washed with MeOH till the solution ran clear (red to colourless). Batches of QD-*(R)*-sSPhos (10 g, 12.21 mmol, >99:1 d.r) were dissolved in MeOH (500 mL) and flushed through the washed AmberLite 12 times. Once complete the solution was concentrated under vacuum to yield H-*(R)*-sSPhos (5.7 g, 11.6 mmol, 95%, >99% ee) as a pale light yellow solid. **Chiral SFC Analysis** (IH-3, 70:30 CO_2:MeOH, 2.5 mL/min, 2.21 min [major], 5.51 min [minor]).

H-*(S)*-sSPhos:

AmberLite IR120H (150 g) was washed with MeOH till the solution ran clear (red to colourless). Batches of QD-*(S)*-sSPhos (10 g, 12.21 mmol, 15:85 d.r) were dissolved in MeOH (500 mL) and flushed through the washed AmberLite 8 times. Once complete the solution was concentrated under vacuum. MeCN (500 ml) was added and sonicated before being concentrated under vacuum, this was repeated twice. MeCN (500 ml) was again added and sonicated to give a milky-white suspension, which was filtered to give enantioenriched H-*(S)*-sSPhos (2.4 g, 4.9 mmol, 40%,1:99 e.r) as a pasty white solid, which was further dried under vacuum to give a free flowing solid. **Chiral SFC Analysis** (IH-3, 70:30 CO_2:MeOH, 2.5 mL/min, 2.21 min [minor], 5.51 min [major]).

Full Procedure: 250 mmol scale using a Radleys® Reactor

QD-*(R/S)*-sSPhos:

Trifluoroacetic anhydride (92 mL, 0.66 mol, 2.63 eq.) was added dropwise to sulfuric acid (18 mL, 0.34 mol, 1.25 eq.), using a dropping funnel (flow rate of 10 mL/min). Once complete this biphasic mixture was stirred at room temperature for 17 h to form a homogenous yellow solution of TFAA-H_2SO_4. *The homogenous solution is typically formed after 4 hours.* SPhos (102.65 g, 0.25 mol) was added portion wise (10g batches every minute) to a solution of TFAA-H_2SO_4 (95 mL, 0.27 mmol, 1.08 eq.) and TFA (150 mL) at a jacket temperature of 0 °C in a 2 L Radleys® reactor, to form a yellow solution, which was then stirred (200 rpm) at a jacket temperature of 20 °C for the 17 h. *Addition of SPhos to this mixture resulted in a minor exotherm, ~5 °C.* The reaction mixture was then concentrated under reduced pressure at a jacket temperature of 60 °C, within the reactor to form a yellow gel. EtOAc (1 L) was added to form a white suspension, which was stirred (200 rpm) for 1 hour, at a jacket temperature of 20 °C. The suspension was removed from the reactor and filtered to yield a paste-like white solid. The solid was further dried under pressure, to give a H-*(rac)*-sSPhos as a free-flowing white powder (79.6 g, 0.16 mol, 65%). *Note: Incomplete extraction of H-(rac)-sSPhos had occurred, thus the remaining product (36.7 g , 0.07 mol, 30%) was isolated by concentrating the filtrate under vacuum, adding EtOAc and filtering, following the same procedure as above. Only the first batch of H-(rac)-sSPhos* (79.6 g, 0.16 mol, 65%) *obtained was carried forward into the subsequent steps.*

H-*(rac)*-sSPhos (79.6 g, 0.16 mol), Quinidine (55.3 g, 0.17 mol, 1.05 eq.) and MeCN (2.90 L) were stirred (200 rpm) under reflux in a 5L Radleys® reactor, at a jacket temperature of 100 °C (Internal reaction temperature ~83 °C). Once the milky-white suspension became a colourless/light yellow solution (25-40 min), the heating and stirring was stopped and the reaction mixture was allowed to slowly cool for 17 h. *Note: The product recrystalizes to the vessel walls*. The filtrate was removed by decanting to leave QD-*(R)*-sSPhos (51.4 g, 63.18 mmol, 39%, 96:04 d.r) remaining as a white powder stuck to the reactor. A small portion of the precipitate was taken and converted to H-sSPhos (method below) and analysed by chiral SFC. **Chiral SFC Analysis** (IH-3, 70:30 CO_2:MeOH, 2.5 mL/min, 2.21 min [major], 5.51 min [minor]). The filtrate was concentrated under vacuum to obtain QD-*(S)*-sSPhos (78.77 g, 96.82 mmol, 61%, 18:82 d.r), as

a light yellow solid. Again a small portion of the precipitate was taken and converted to H-sSPhos (method below) and analysed by chiral SFC. **Chiral SFC Analysis** (IH-3, 70:30 CO_2:MeOH, 2.5 mL/min, 2.21 min [minor], 5.51 min [major]). *The d.r. of QD-sSPhos was inferred from the measured e.r. of H-sSPhos.*

AmberLite IR120H (1.5 g) was washed with MeOH till the solution ran clear. QD-sSPhos (50 mg, 0.06 mmol) was dissolved in MeOH (1 mL) and flushed through the washed AmberLite 12 times. Once complete the solution was concentrated under vacuum to yield H-sSPhos, as a light yellow solid.

<u>H-*(R)*-sSPhos:</u>

MeCN (2.90 L) was added back to the reactor containing the recrystallized QD-*(R)*-sSPhos (51.4 g, 63.18 mmol, 96:04 d.r) and again the solution was stirred under reflux, at a jacket temperature of 100 °C (Internal reaction temperature ~83 °C). Once the milky-white suspension became a colourless/light yellow solution (25-40 min), the heating was stopped and the reaction mixture was allowed to cool to r.t and stirred for 17 h. The filtrate was again decanted out of the reactor, before more MeCN (50 ml) was added and decanted off. *Leaving the product recrystalized as a white-solid on the vessel walls*. XRPD analysis of a sample of this material was carried out (attached as "XRPD1"). The precipitated product was then extracted off the walls of the reactor, by adding MeOH (200 mL) and refluxing at a jacket temperature of 80 °C and stirring (350 rpm) to form a solution of the product in MeOH. This solution was removed from the reactor and concentrated under vacuum giving a light yellow/white solid, QD-*(R)*-sSPhos (41.12 g, 50.54 mmol, 80%, >99:1 d.r). XRPD analysis of a sample of this material was carried out, indicating it is amorphous (attached as "XRPD2").AmberLite IR120H (300 g) was washed with MeOH till the solution ran clear (red to colourless). Batches of QD-*(R)*-sSPhos (10 g, 12.21 mmol, >99:1 d.r) were dissolved in MeOH (500 mL) to give a light yellow/colourless solution and flushed through the washed AmberLite 12 times. Once complete the solution was concentrated under vacuum to yield H-*(R)*-sSPhos (5.7 g, 11.6 mmol, 95%, >99% ee) as a pale light yellow solid. **Chiral SFC Analysis** (IH-3, 70:30 CO_2:MeOH, 2.5 mL/min, 2.21 min [major], 5.51 min [minor]).

<u>H-*(S)*-sSPhos:</u>

AmberLite IR120H (150 g) was washed with MeOH till the solution ran clear (red to colourless). Batches of QD-*(S)*-sSPhos (10 g, 12.21 mmol, 18:82 d.r) were dissolved in MeOH (500 mL) and flushed through the washed AmberLite 8 times. Once complete the solution was concentrated under vacuum. MeCN (500 ml) was added and sonicated before being concentrated under vacuum, this was repeated twice. MeCN (500 ml) was again added and sonicated to give a a milky-white suspension, which was filtered to give enantioenriched H-*(S)*-sSPhos (2.4 g, 4.9 mmol, 40%,1:99 e.r) as a pasty white solid, which was dried under vacuum to give a free flowing solid. **Chiral SFC Analysis** (IH-3, 70:30 CO_2:MeOH, 2.5 mL/min, 2.21 min [minor], 5.51 min [major]).

sSPhos Characterization Data:

QD-(R)-sSPhos (>99:01): **^{1}H NMR** (700 MHz, $CDCl_3$) δ 11.34 (s, 1H), 8.74 (d, *J* = 4.5 Hz, 1H), 8.00 (dd, *J* = 32.5, 9.0 Hz, 2H), 7.69 (d, *J* = 4.5 Hz, 1H), 7.53 (ddd, *J* = 7.7, 3.6, 1.5 Hz, 1H), 7.46 – 7.35 (m, 3H), 7.30 (dd, *J* = 9.2, 2.6 Hz, 1H), 7.28 – 7.22 (m, 1H), 6.68 (d, *J* = 8.9 Hz, 1H), 6.54 (d, *J* = 4.5 Hz, 1H), 6.04 (ddd, *J* = 17.4, 10.5, 7.3 Hz, 1H), 5.83 (br s, 1H), 5.28 – 5.19 (m, 2H), 4.25 (dd, *J* = 12.7, 8.3 Hz, 1H), 3.94 (s, 3H), 3.70 (s, 3H), 3.53 (t, *J* = 11.9 Hz, 1H), 3.49 (m, 1H), 3.39 (s, 3H), 3.36 – 3.32 (m, 2H), 2.59 (q, *J* = 8.8 Hz, 1H), 2.43 (m, 1H), 2.01 – 1.90 (m, 3H), 1.77 – 1.56 (m, 7H), 1.44 (m, 4H), 1.23 – 0.62 (m, 12H). **^{13}C NMR** (176 MHz, $CDCl_3$) δ 159.6, 158.9, 155.2, 146.8, 144.5, 144.0, 143.8, 141.5 (d, *J* = 27.9 Hz), 136.4, 132.4, 132.3 (m), 131.2, 131.0, 129.8 (m), 128.7, 126.9, 126.6, 125.9, 122.8, 118.8, 117.6, 105.0, 100.5, 66.4, 61.0, 60.2, 57.2, 55.5, 49.4, 48.5, 37.5, 35.4, 32.3, 29.6 (m), 29.0 (m), 27.6, 27.3 (m), 27.0 (d, *J* = 10.0 Hz), 26.7 (d, *J* = 12.0 Hz), 26.2, 25.9 (m),

23.4, 17.9. [31]**P NMR** (162 MHz, $CDCl_3$) δ -8.73. *Cation exchange to H-sSPhos was performed before* **Chiral SFC Analysis** (IH-3, 70:30 CO_2:MeOH, 2.5 mL/min, 2.21 min [major], 5.51 min [minor]).

contains ~12% dihydroquinidine

QD-(S)-sSPhos (18:82) : ^{1}**H NMR** (700 MHz, $CDCl_3$) δ 11.16 (s, 1H), 8.68 (dd, *J* = 4.5, 3.4 Hz, 1H), 8.11 (d, *J* = 8.7 Hz, 1H), 7.86 (dd, *J* = 9.1, 7.4 Hz, 1H), 7.65 (dd, *J* = 4.5, 0.7 Hz, 1H), 7.61 – 7.53 (m, 1H), 7.41 – 7.34 (m, 2H), 7.28 – 7.12 (m, 2H), 6.62 (d, *J* = 8.8 Hz, 1H), 6.40 (d, *J* = 3.1 Hz, 1H), 6.28 (s, 1H), 5.99 (ddd, *J* = 17.4, 10.4, 7.2 Hz, 1H), 5.28 – 5.05 (m, 1H), 4.23 (ddd, *J* = 13.2, 8.5, 2.4 Hz, 1H), 3.90 (d, *J* = 7.2 Hz, 3H), 3.62 (s, 2H), 3.46-3.42 (m, 3H), 3.39 – 3.30 (m, 2H), 3.23 (m, 1H), 3.11 (br s, 1H), 2.51 (m, 1H), 2.35 (ddt, *J* = 13.6, 9.8, 1.9 Hz, 1H), 2.06 (m, 2H), 1.94-1.82 (m, 2H), 1.80 – 1.36 (m, 13H), 1.33 – 0.84 (m, 12H). ^{13}**C NMR** (176 MHz, $CDCl_3$) δ 163.0 (d, *J* = 35.0 Hz), 159.8, 159.7, 158.5, 155.6, 155.6, 155.5, 147.0, 144.5, 144.4, 143.8, 141.7, 141.7, 141.6 (d, *J* = 31.1 Hz), 135.4 (d, *J* = 15.3 Hz), 132.4 (d, *J* = 3.4 Hz), 132.0 (d, *J* = 6.4 Hz), 132.0, 131.3, 130.7, 130.6, 129.3, 129.3, 128.1, 128.1, 126.7, 126.5 (d, *J* = 6.3 Hz), 126.4, 125.6, 125.6, 122.4, 118.7, 117.6, 117.5, 116.4, 116.0, 105.0, 104.7, 100.2, 66.7, 66.5, 61.0, 61.0, 60.2, 60.1, 56.7, 56.7, 55.4, 50.3, 49.5, 49.4, 48.5, 37.4, 35.4 (d, *J* = 13.0 Hz), 35.2 (m), 32.7 (d, *J* = 10.8 Hz), 30.2 (d, *J* = 18.1 Hz), 29.6 (m), 29.5, 29.4 (m), 29.3 (m) 29.1 (d, *J* = 7.7 Hz), 27.7 (m), 27.6 (m), 27.5 (m), 27.54 (m), 27.1 (m), 27.1 (dd, *J* = 10.6, 6.1 Hz), 27.0, 26.4 (m) 26.3, 26.1, 25.1, 24.2, 23.9, 23.4, 18.0, 11.5, 1.9. ^{31}**P NMR** (162 MHz, $CDCl_3$) δ -8.90. *Cation exchange to H-sSPhos was performed before* **Chiral SFC Analysis** (IH-3, 70:30 CO_2:MeOH, 2.5 mL/min, 2.21 min [minor], 5.51 min [major]).

2'-(dicyclohexylphosphaneyl)-2,6-dimethoxy-[1,1'-biphenyl]-3-sulfonic acid (H-*(rac)*-sSPhos): ^{1}**H NMR** (700 MHz, MeOD) δ 8.07 (d, *J* = 8.9 Hz, 1H), 7.99 (dd, *J* = 11.0, 8.1 Hz, 1H), 7.91 (t, *J* = 7.2 Hz, 1H), 7.76 (t, *J* = 6.7 Hz, 1H), 7.69 (d, *J* = 4.6 Hz, 1H), 7.05 (d, *J* = 8.9 Hz, 1H), 3.82 (s, 1H), 3.40 (s, 3H), 3.02 (q, *J* = 11.6 Hz, 3H), 2.52 (q, *J* = 12.2 Hz, 1H), 2.08 (m, 1H), 2.00 – 1.92 (m, 1H), 1.92 – 1.86 (m, 2H), 1.79 (m, 3H), 1.72 – 1.59 (m, 4H), 1.57 – 1.24 (m, 6H), 1.08 (m, 3H). ^{13}**C NMR** (176 MHz, MeOD) δ 159.6 (d, *J* = 0.8 Hz), 154.0, 140.2 (d, *J* = 5.7 Hz), 134.4 (d, *J* = 9.0 Hz), 133.9 (d, *J* = 3.1 Hz), 132.9 (d, *J* = 10.1 Hz), 131.7, 131.1, 128.6 (d, *J* = 12.0 Hz), 121.6 (d, *J* = 4.5 Hz), 113.4 (d, *J* = 80.0 Hz), 106.7, 28.8 (d, *J* = 43.6 Hz), 27.2 (d, *J* = 43.5 Hz), 26.5 (m), 26.4 (d, *J* = 2.2 Hz), 25.7 (d, *J* = 6.7 Hz), 25.6 (d, *J* = 6.1 Hz), 25.4 (d, *J* = 14.0 Hz), 25.1 (d, *J* = 13.7 Hz), 24.8 (d, *J* = 2.1 Hz), 24.8 (d, *J* = 2.2 Hz), 24.7 (d, *J* = 4.1 Hz). ^{31}**P NMR** (162 MHz, MeOD) δ 17.3 (br t).

H-*(R)*-sSPhos

Chiral SFC Analysis: (IH-3, 70:30 CO_2:MeOH, 2.5 mL/min, 2.21 min [major], 5.51 min [minor])

$[\alpha]^{25}_{D}$ = - 107.4 (c 0.12, MeOH)). Obtained from recrystallized H-*(R)*-sSPhos (>99% ee).

H-(S)-sSPhos

Chiral SFC Analysis: (IH-3, 70:30 CO_2:MeOH, 2.5 mL/min, 2.21 min [minor], 5.51 min [major])

$[\alpha]\frac{25}{D}$ = + 104.9 (c 0.12, MeOH)). Obtained from recrystallized H-*(S)*-sSPhos (98% ee).

Enantioselective Suzuki-Miyaura Coupling

(R)-6-fluoro-6'-methyl-[1,1'-biphenyl]-2,2'-diol

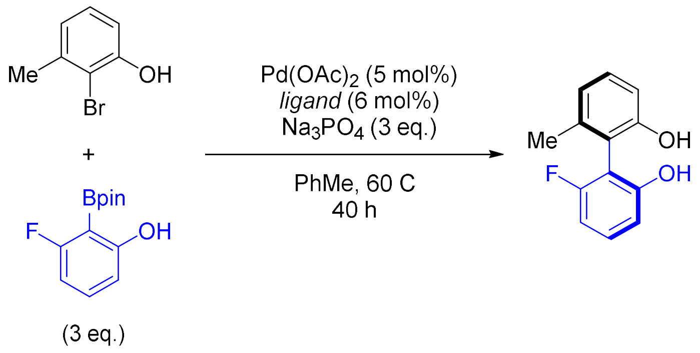

1 mmol scale:

3-methyl-2-bromophenol (187 mg, 1 mmol), 2-hydroxy 6-fluorophenylboronic acid pinacol ester (710 mg, 3 mmol), $Pd(OAc)_2$ (11 mg, 0.05 mmol, 5 mol%), H-*(R)*-sSPhos (29.4 mg, 0.06 mmol, 6 mol%, >99%ee) and Na_3PO_4 (490 mg, 3 mmol) were added to a 20 mL crimp vial. After 10 evacuation-backfill cycles with nitrogen, toluene (5 mL) was added. The reaction mixture was stirred at 60 °C, monitoring the reaction till it was over. The solvent was removed under a vacuum and the product was purified by column chromatography (10–20% EtOAc: petrol) to yield the title compound, (159.1 mg, 0.73 mmol, 73%, 94% ee). **^{1}H NMR** (700 MHz, $CDCl_3$) δ 7.32 (td, *J* = 8.3, 6.5 Hz, 1H), 7.26 (t, *J* = 7.9 Hz, 1H)*, 6.94 (dt, *J* = 7.6, 0.9 Hz, 1H), 6.90 – 6.86 (m, 2H), 6.80 (td, *J* = 8.5, 1.0 Hz, 1H), 5.02 (s, 1H), 4.79 (s, 1H), 2.09 (s, 3H). **^{13}C NMR** (176 MHz, $CDCl_3$) δ 160.6 (d, *J* = 246.5 Hz), 154.8 (d, *J* = 5.8 Hz), 154.0, 140.0, 130.9 (d, *J* = 10.3 Hz), 130.6, 122.8, 115.0, 113.4, 111.6 (d, *J* = 3.3 Hz), 109.3 (d, *J* = 20.9 Hz), 108.0 (d, *J* = 22.2 Hz), 19.8. **^{19}F NMR** (376 MHz, $CDCl_3$) δ -112.9. **Chiral HPLC Analysis** (SC-3, Hexane: IPA 97:03, 1.25 mL min^{-1} , 40 °C) tR = 14.72 (major), 18.63 (minor) minutes.

The same reaction was repeated but using H-*(S)*-sSPhos (29.4 mg, 0.06 mmol, 6 mol%, 98%ee), to give the titled compound (159.1 mg, 0.73 mmol, 73%, 94% ee). **Chiral HPLC Analysis** (SC-3, Hexane: IPA 97:03, 1.25 mL min^{-1} , 40 °C) tR = 14.72 (minor), 18.63 (major) minutes.

Data in accordance with literature.

0.1 mmol scale:

3-methyl-2-bromophenol (18.7 mg, 0.1 mmol), 2-hydroxy 6-fluorophenylboronic acid pinacol ester (71.0 mg, 3 mmol), $Pd(OAc)_2$ (1.1 mg, 0.05 mmol, 5 mol%), H-*(R)*-sSPhos (2.94 mg, 0.006 mmol, 6 mol%, >99%ee) and Na_3PO_4 (49.0 mg, 3 mmol) were added to a 4 mL crimp vial. After 5 evacuation-backfill cycles with nitrogen, toluene (0.5 mL) was added. The reaction mixture was stirred at 60 °C, for 40 h. The solvent was removed under a stream of air and the product was purified by column chromatography (10–20% EtOAc: petrol) to yield the title compound, (17.7 mg, 0.081 mmol, 81%, 94% ee).

The same reaction was repeated but using Na-*(R)*-sSPhos (2.94 mg, 0.006 mmol 6 mol%, >99%ee SFC separated), to give the titled compound (17.9 mg, 0.082 mmol, 82%, 94% ee).

The same reaction was repeated but using QD-*(R)*-sSPhos (4.89 mg, 0.006 mmol, 6 mol%, >99%de), to give the titled compound (14.8 mg, 0.068 mmol, 68%, 96% ee).

^{1}H-NMR ($CDCl_3$): (1S,2R,4S,5R)-2-((S)-hydroxy(6-methoxyquinolin-4-yl)methyl)-5-vinylquinuclidin-1-ium 2’ (R)-(dicyclohexylphosphaneyl)-2,6-dimethoxy-[1,1'-biphenyl]-3-sulfonate (QD-*(R)*-sSPhos)

contains ~12% dihydroquinidine

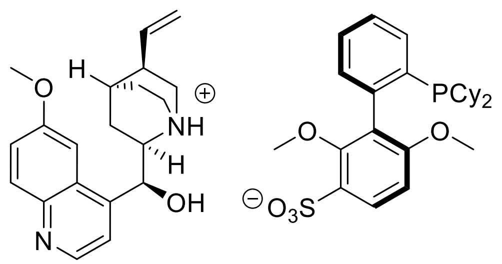

***QD-(R)*-sSPhos (>99:1)**

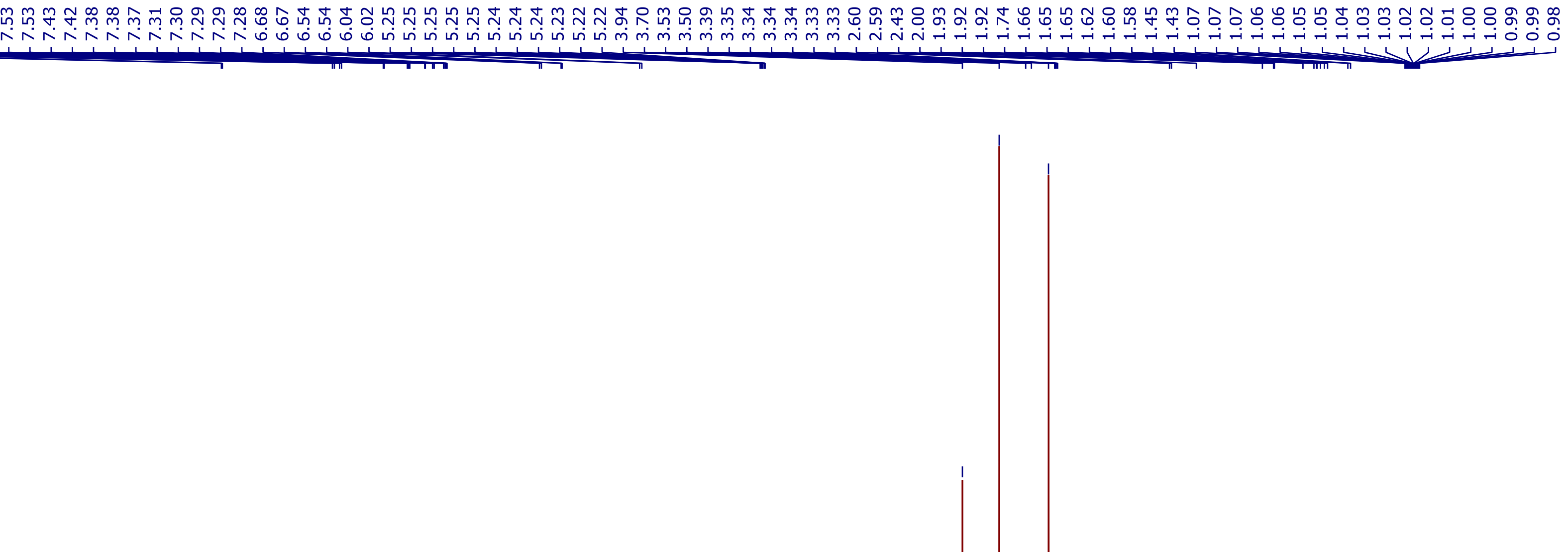
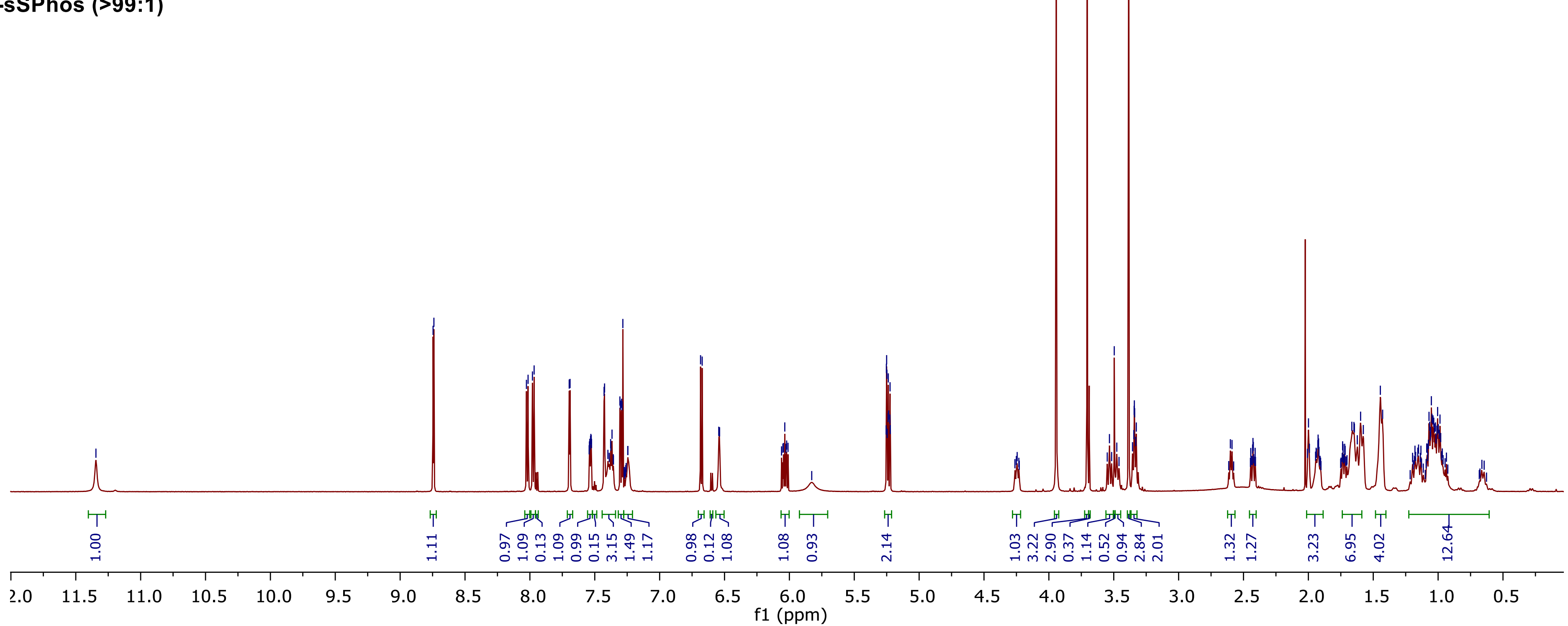

^{13}C-NMR ($CDCl_3$): (1S,2R,4S,5R)-2-((S)-hydroxy(6-methoxyquinolin-4-yl)methyl)-5-vinylquinuclidin-1-ium 2’ (R)-(dicyclohexylphosphaneyl)-2,6-dimethoxy-[1,1'-biphenyl]-3-sulfonate (QD-*(R)*-sSPhos)

contains ~12% dihydroquinidine

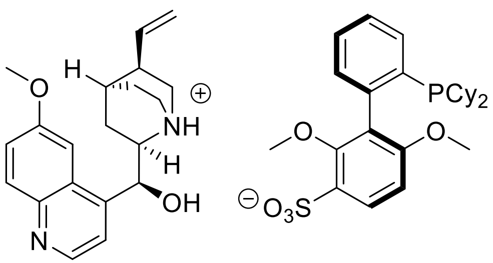

***QD-(R)*-sSPhos (>99:1)**

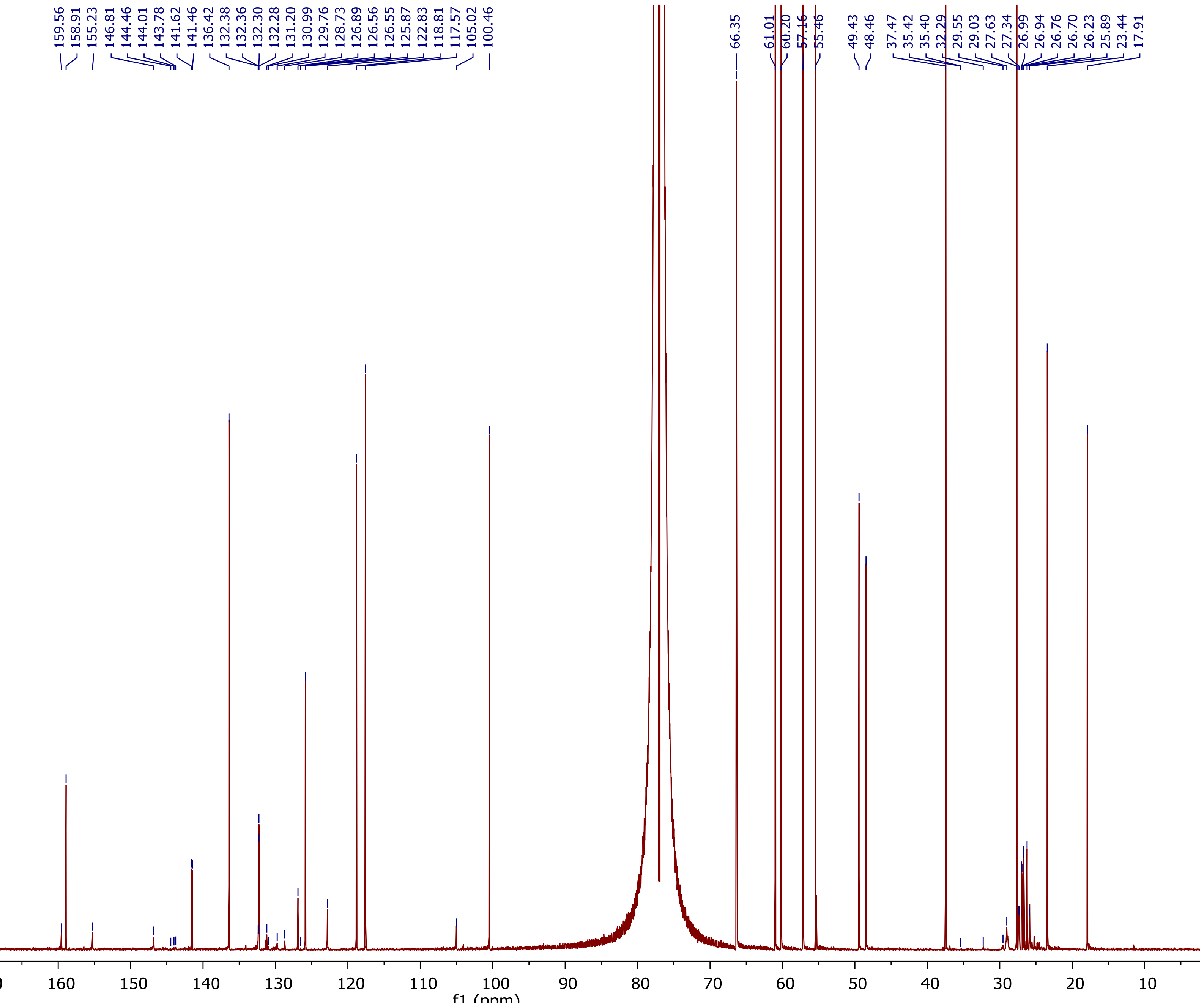

^{31}P-NMR ($CDCl_3$): (1S,2R,4S,5R)-2-((S)-hydroxy(6-methoxyquinolin-4-yl)methyl)-5-vinylquinuclidin-1-ium 2’ (R)-(dicyclohexylphosphaneyl)-2,6-dimethoxy-[1,1'-biphenyl]-3-sulfonate (QD-*(R)*-sSPhos)

contains ~12% dihydroquinidine

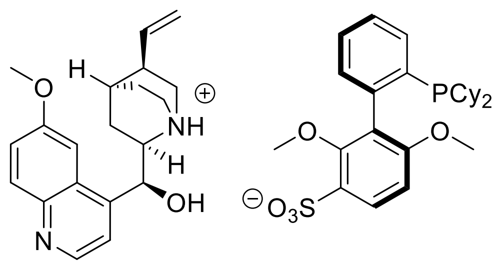

***QD-(R)*-sSPhos (>99:1)**

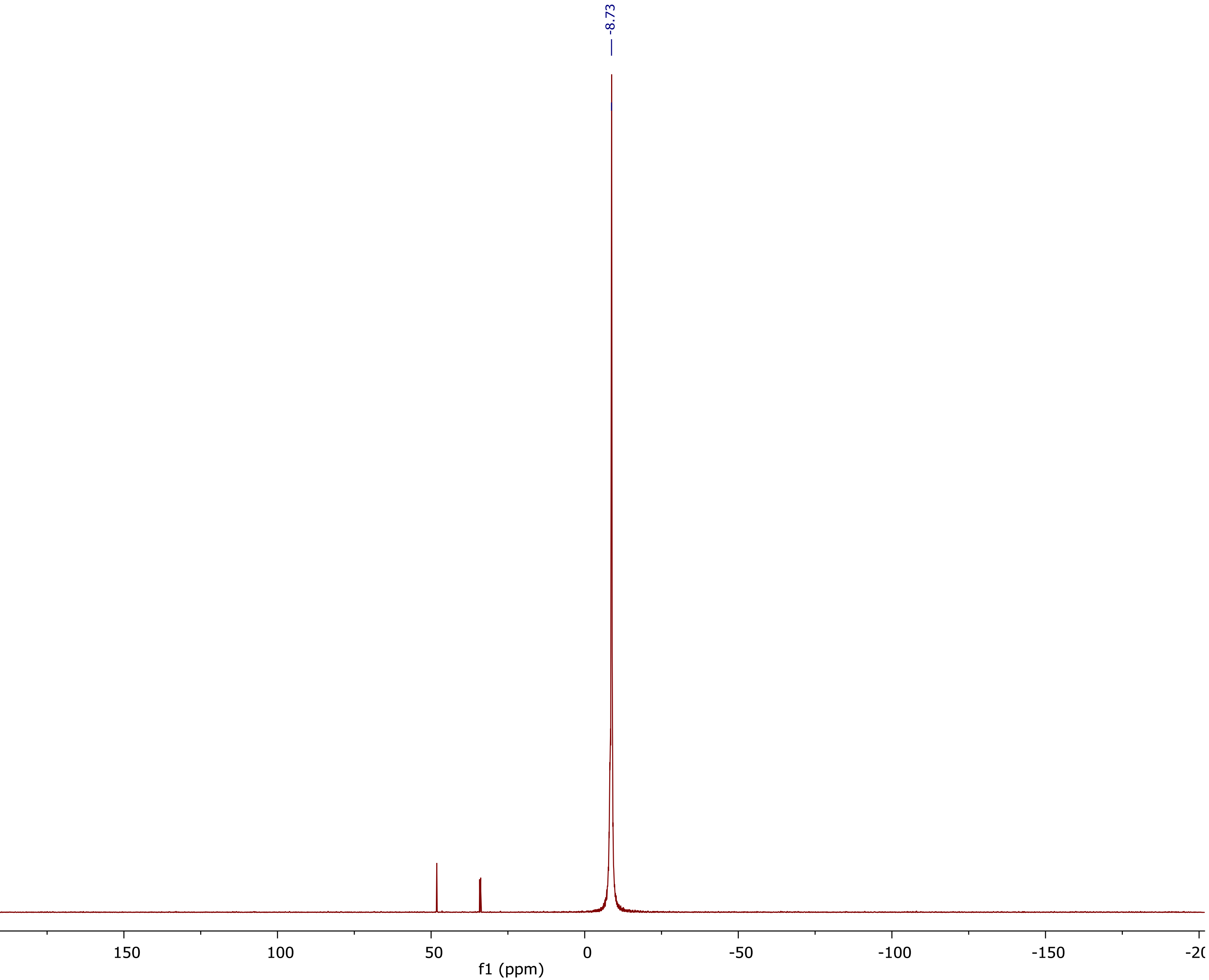

^{1}H-NMR ($CDCl_3$): (1S,2R,4S,5R)-2-((S)-hydroxy(6-methoxyquinolin-4-yl)methyl)-5-vinylquinuclidin-1-ium 2’ (S)-(dicyclohexylphosphaneyl)-2,6-dimethoxy-[1,1'-biphenyl]-3-sulfonate (QD-*(S)*-sSPhos)

contains ~12% dihydroquinidine

contains ~18% QD-(R)-sSPhos

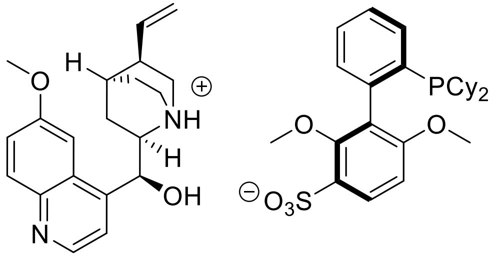

QD-(S)-sSPhos (18:82)

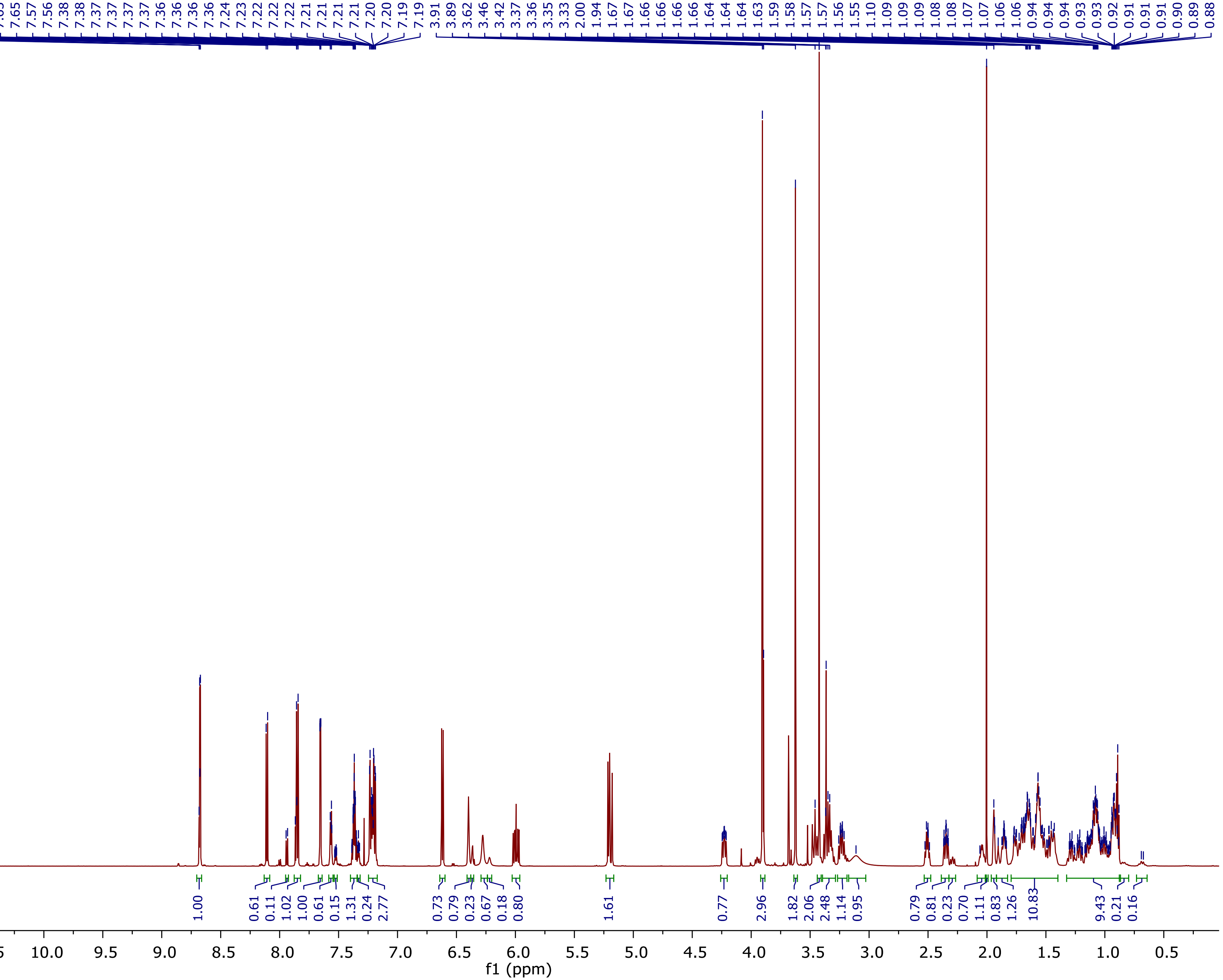

^{13}C-NMR ($CDCl_3$): (1S,2R,4S,5R)-2-((S)-hydroxy(6-methoxyquinolin-4-yl)methyl)-5-vinylquinuclidin-1-ium 2' (S)-(dicyclohexylphosphaneyl)-2,6-dimethoxy-[1,1'-biphenyl]-3-sulfonate (QD-*(S)*-sSPhos)

contains ~12% dihydroquinidine

contains ~18% QD-(R)-sSPhos

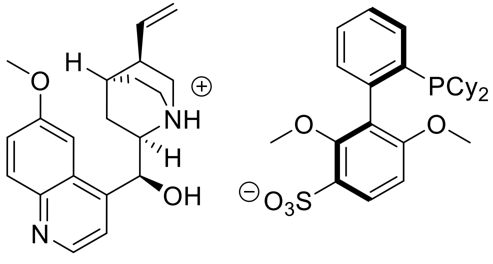

***QD-(S)*-sSPhos (18:82)**

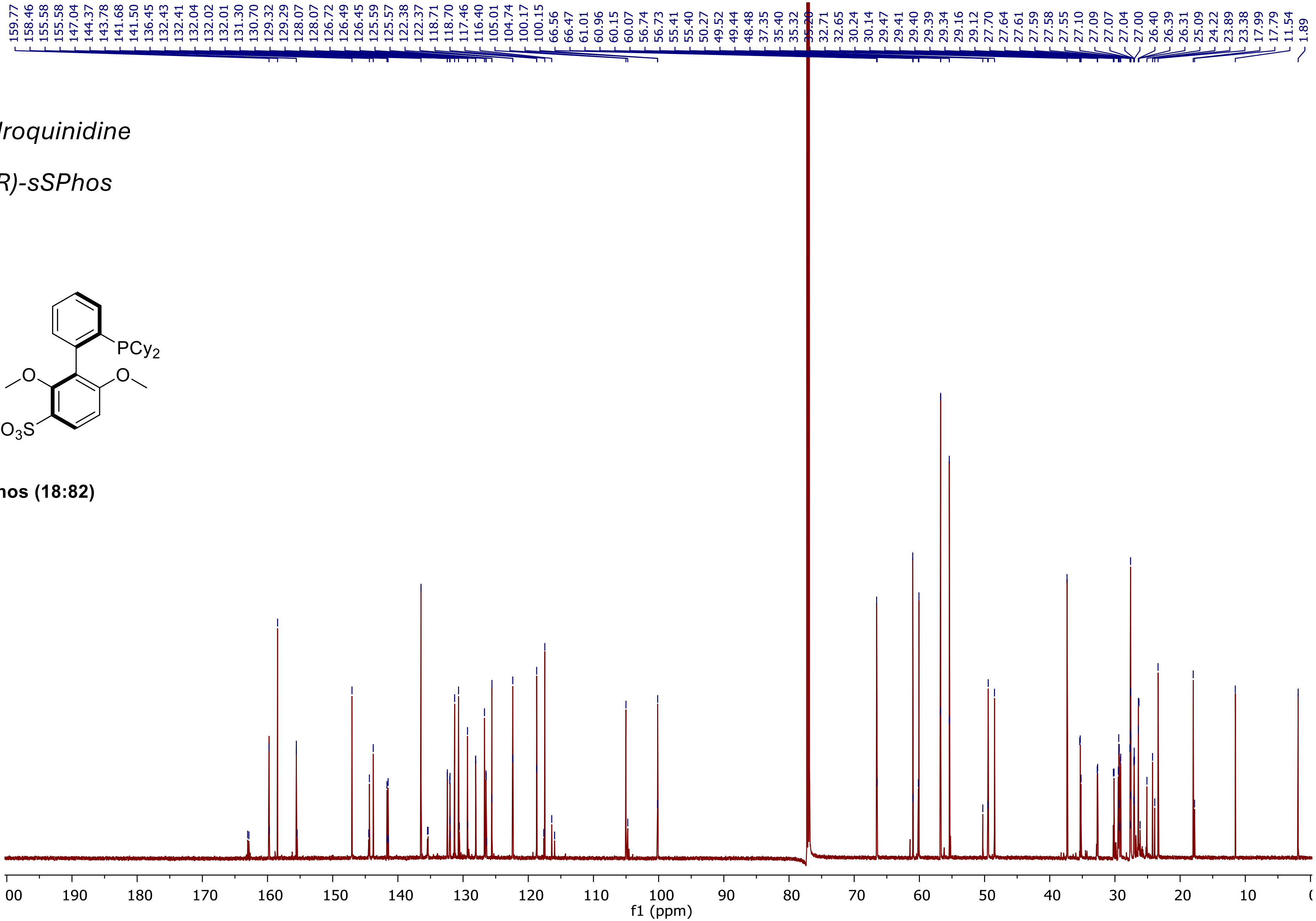

^{31}P-NMR ($CDCl_3$): (1S,2R,4S,5R)-2-((S)-hydroxy(6-methoxyquinolin-4-yl)methyl)-5-vinylquinuclidin-1-ium 2' (S)-(dicyclohexylphosphaneyl)-2,6-dimethoxy-[1,1'-biphenyl]-3-sulfonate (QD-*(S)*-sSPhos)

contains ~12% dihydroquinidine

contains ~18% QD-(R)-sSPhos

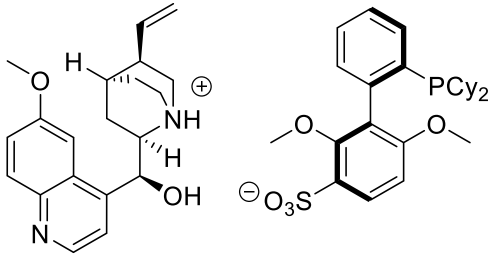

***QD-(S)*-sSPhos (18:82)**

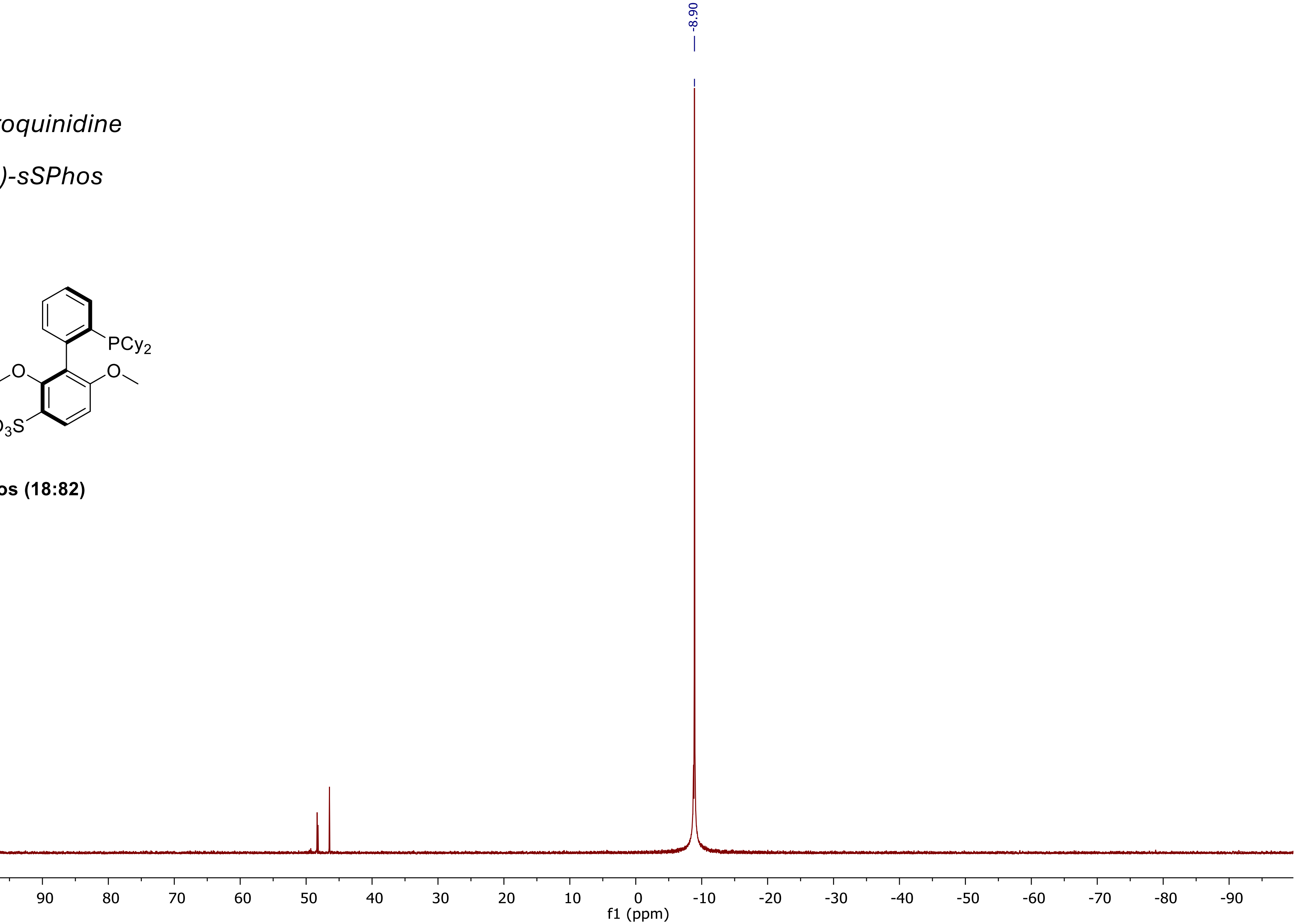

¹H-NMR (MeOD): *(rac)*-2'-(dicyclohexylphosphaneyl)-2,6-dimethoxy-[1,1'-biphenyl]-3-sulfonic acid (H-*(rac)*-sSPhos)

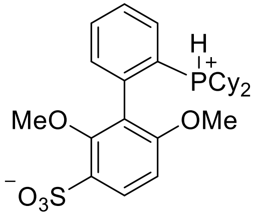

H-*(rac)*-sSPhos

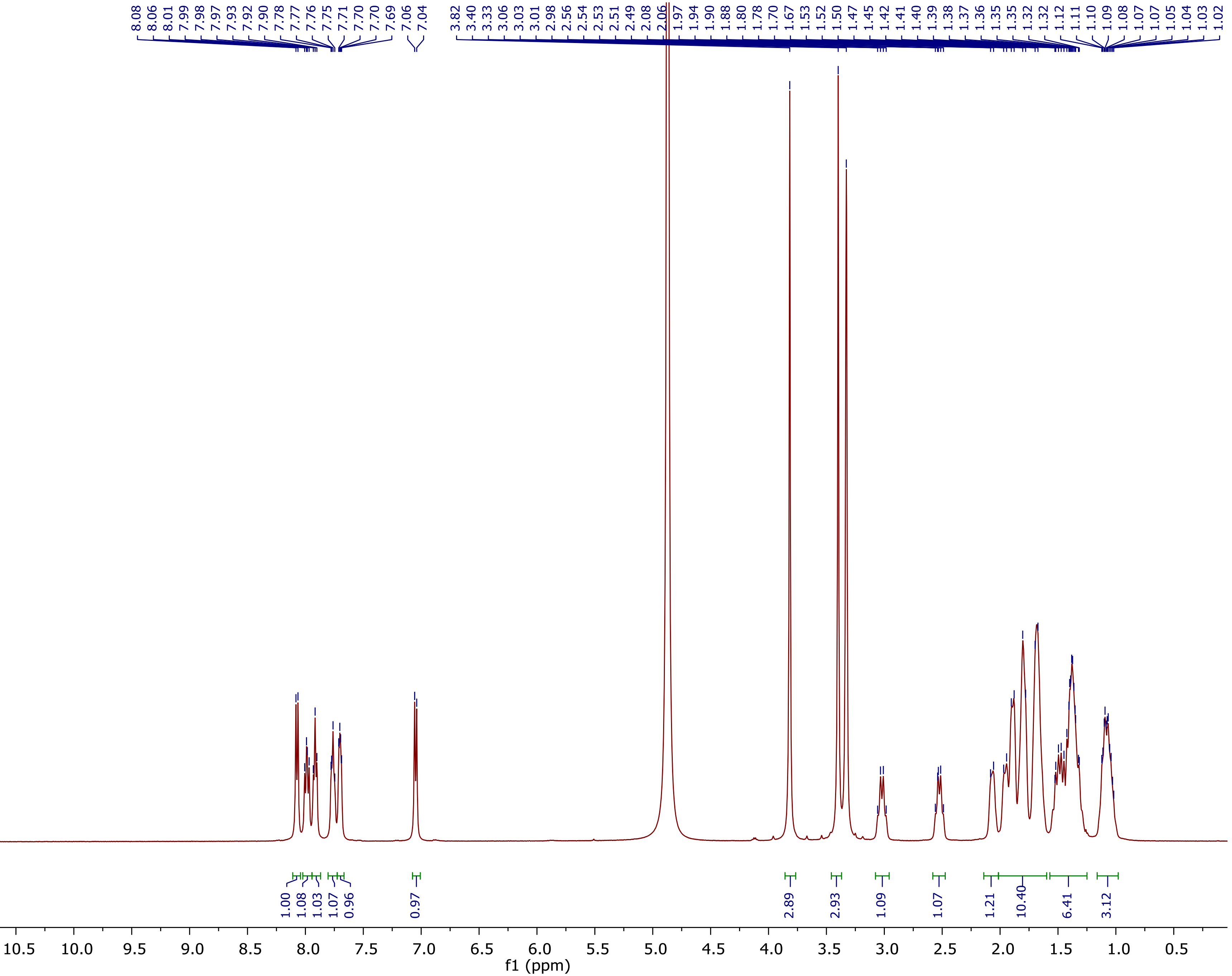

^{1}H-NMR (MeOD): *(R)*-2'-(dicyclohexylphosphaneyl)-2,6-dimethoxy-[1,1'-biphenyl]-3-sulfonic acid (H-*(R)*-sSPhos)

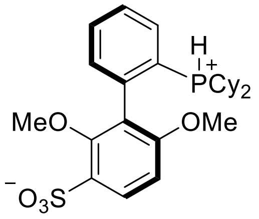

H-*(R)*-sSPhos

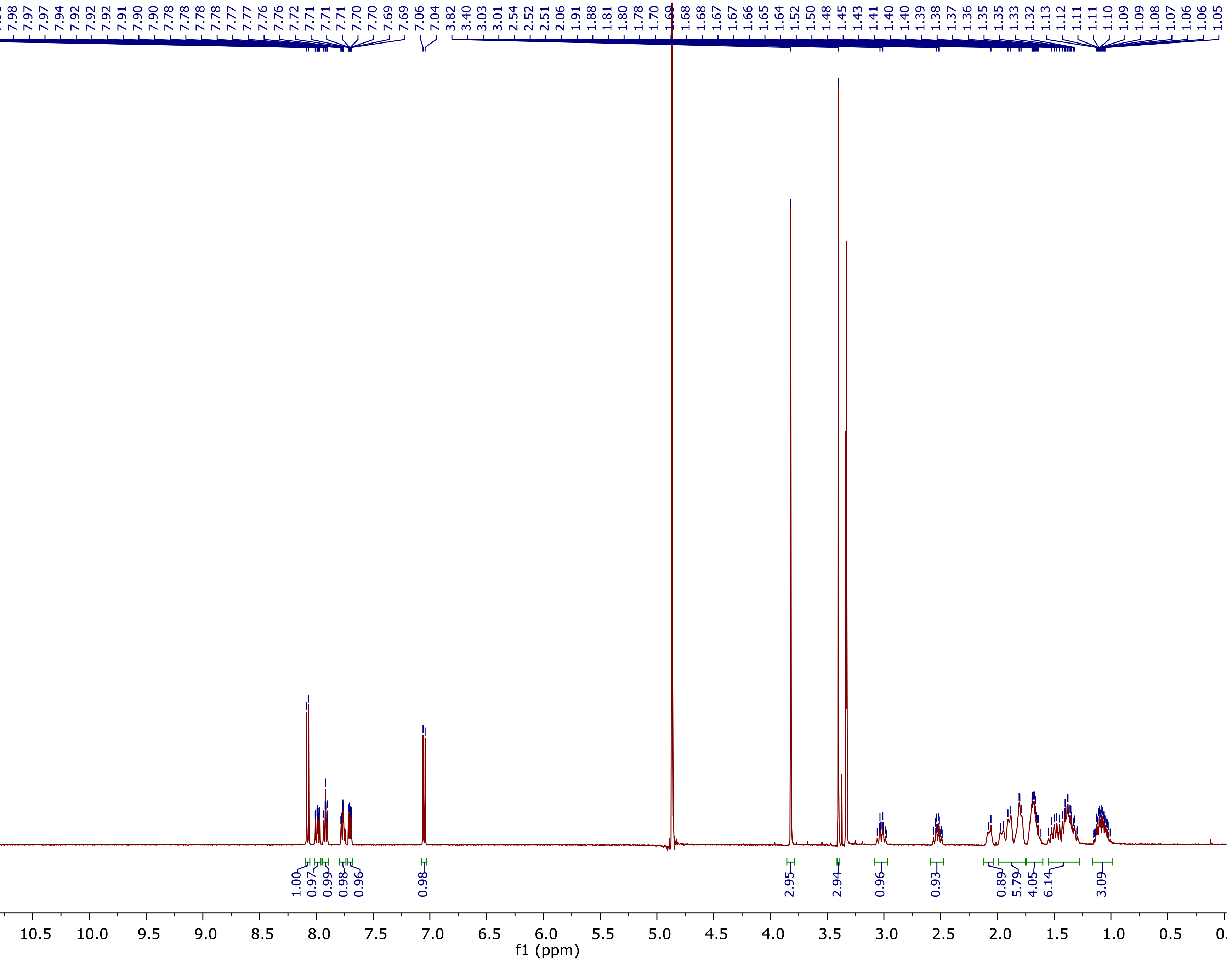

^{1}H-NMR (MeOD): *(S)*-2'-(dicyclohexylphosphaneyl)-2,6-dimethoxy-[1,1'-biphenyl]-3-sulfonic acid (H-*(S)*-sSPhos)

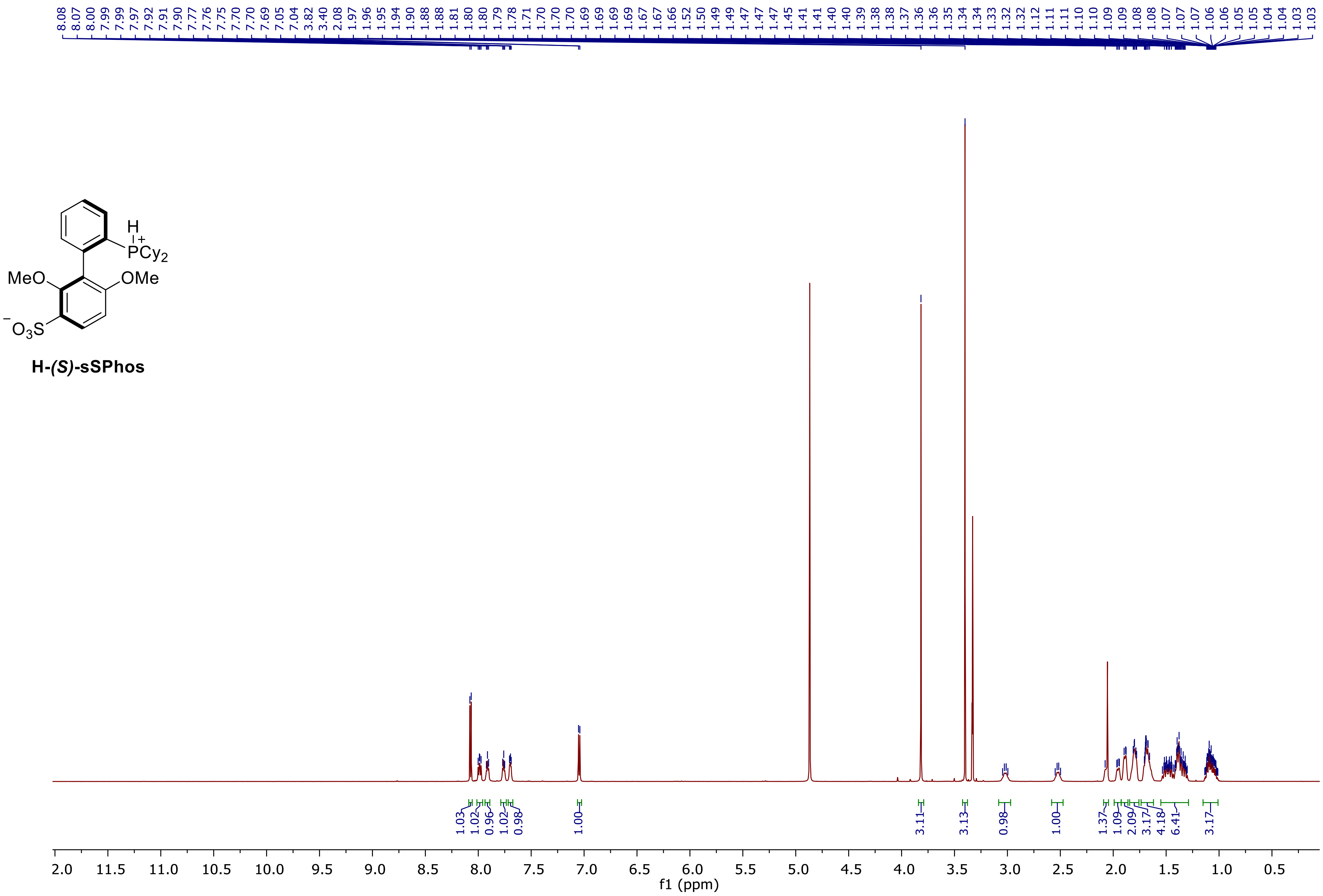

^{13}C-NMR (MeOD): 2'-(dicyclohexylphosphaneyl)-2,6-dimethoxy-[1,1'-biphenyl]-3-sulfonic acid

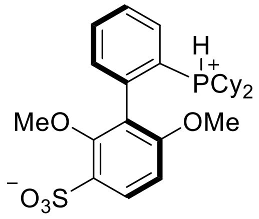

H-*(R)*-sSPhos

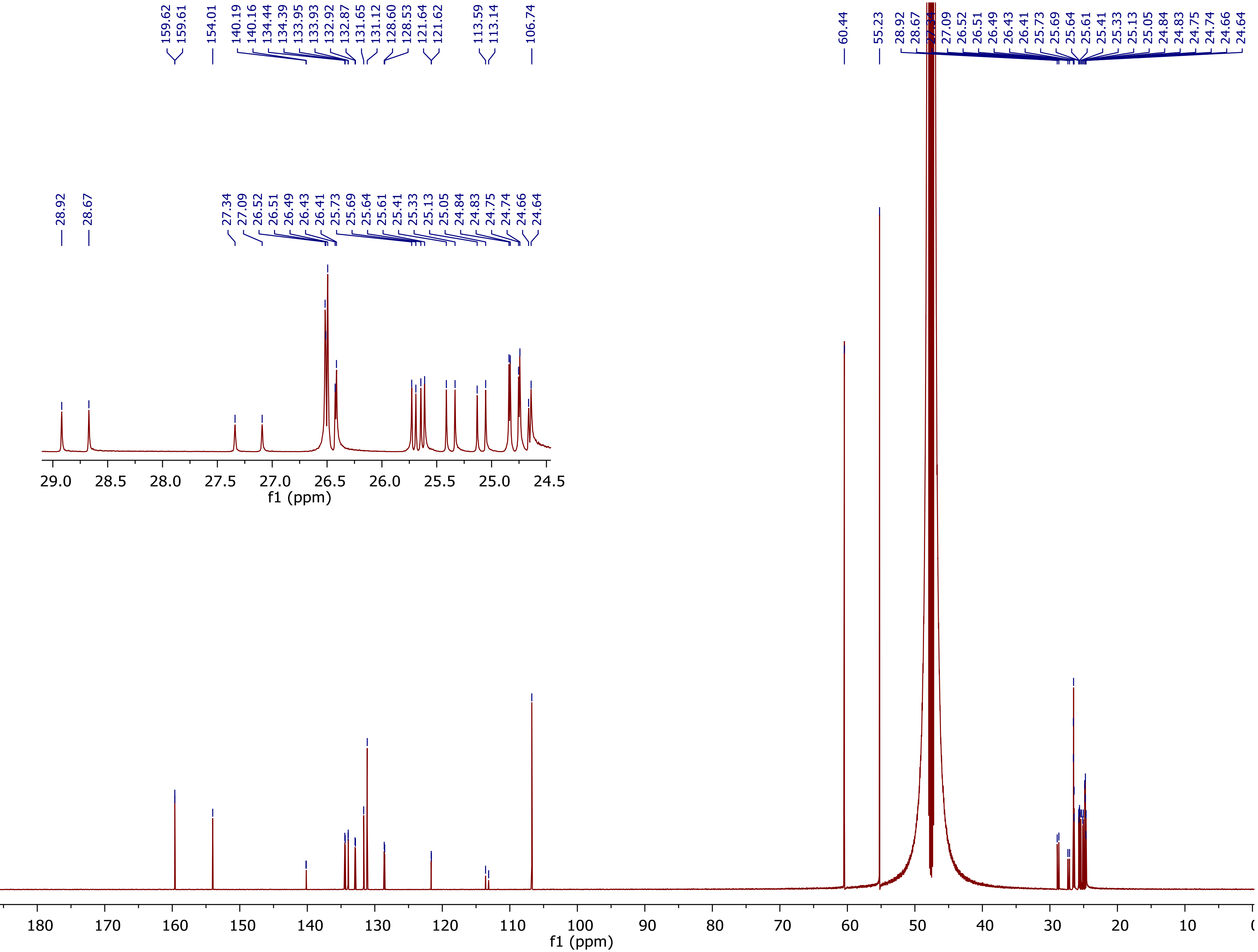

^{31}P-NMR (MeOD): *(R)*-2'-(dicyclohexylphosphaneyl)-2,6-dimethoxy-[1,1'-biphenyl]-3-sulfonic acid

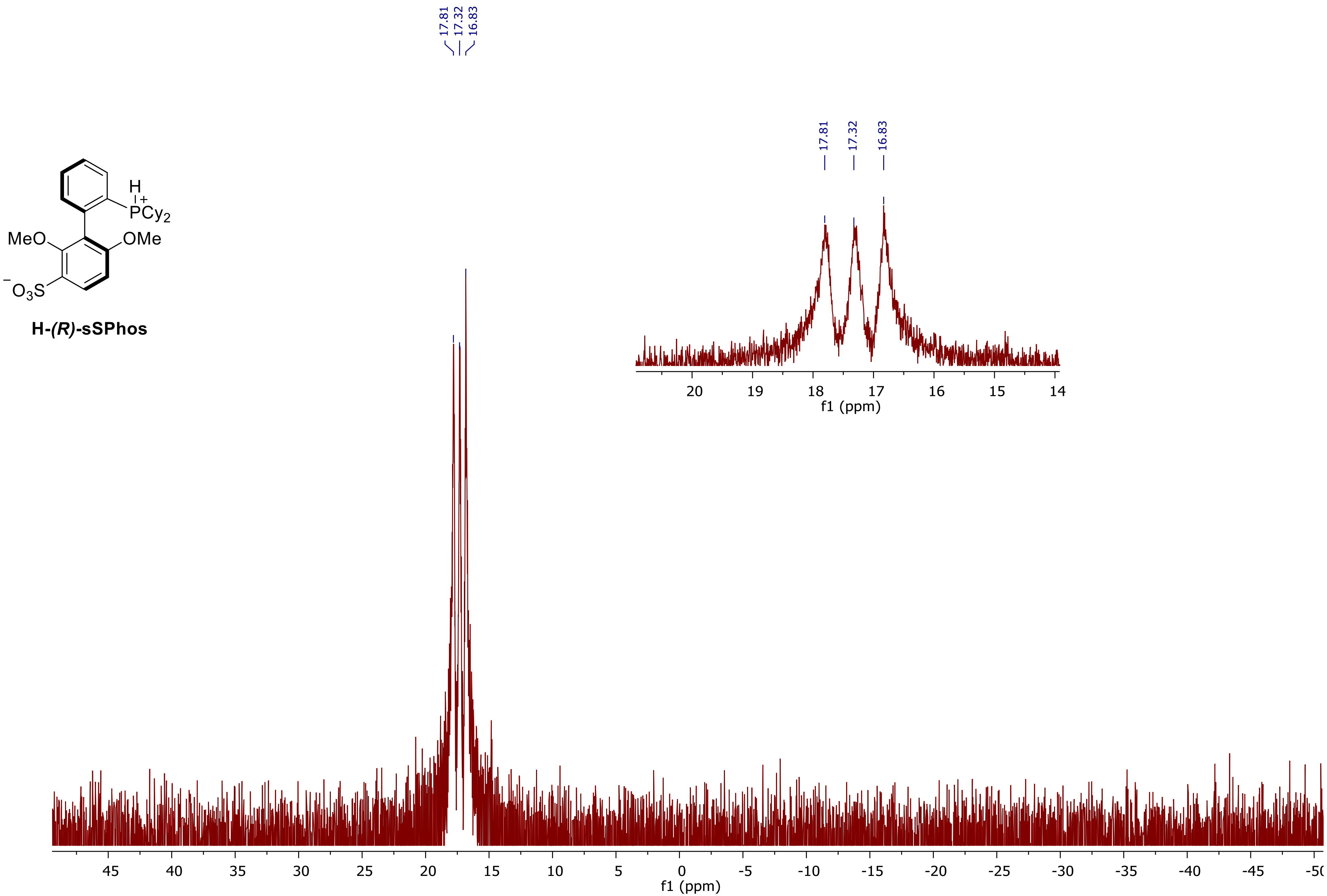

¹H-NMR ($CDCl_3$): (R)-6-fluoro-6'-methyl-[1,1'-biphenyl]-2,2'-diol

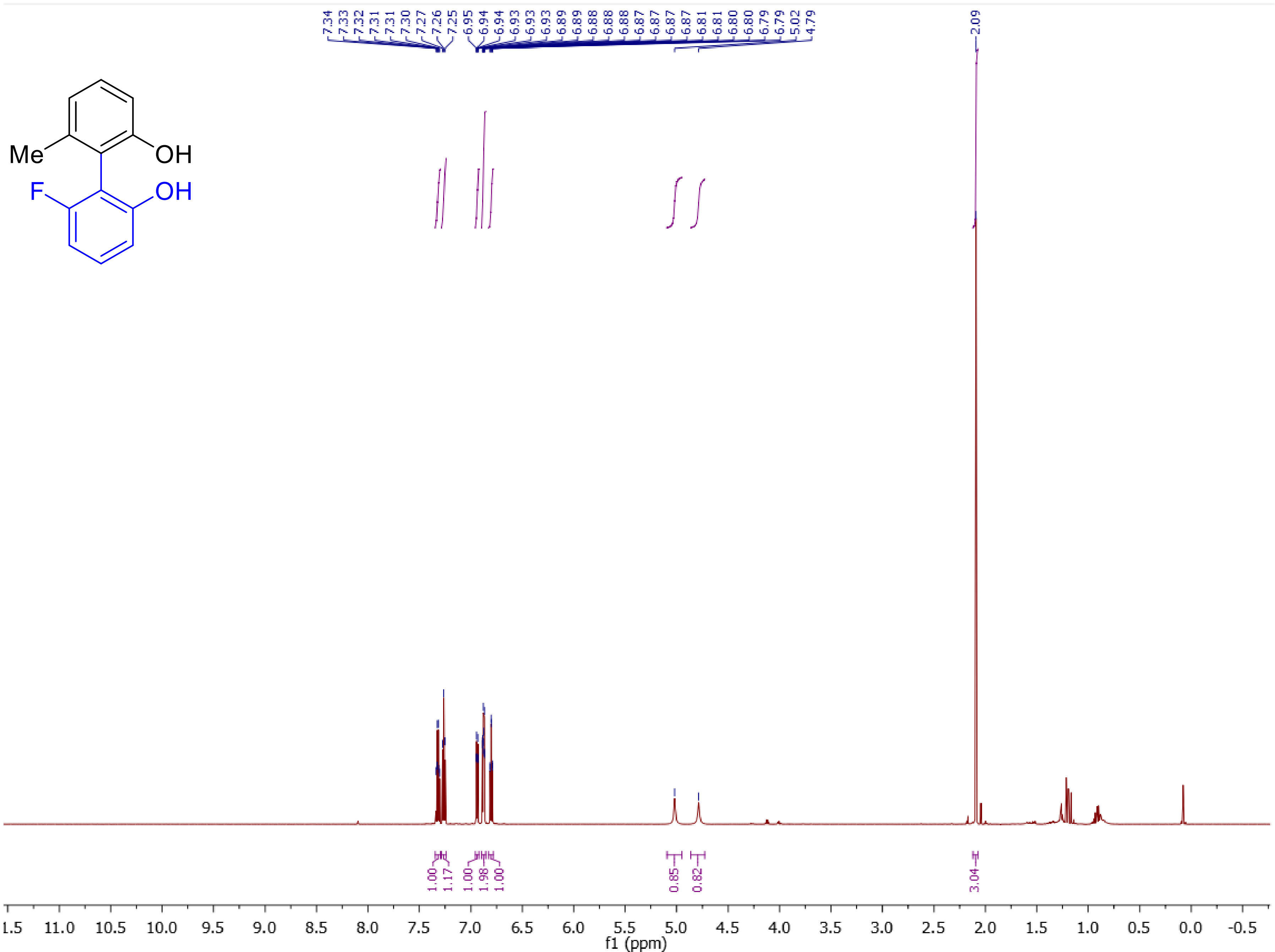

^{13}C-NMR ($CDCl_3$): (R)-6-fluoro-6'-methyl-[1,1'-biphenyl]-2,2'-diol

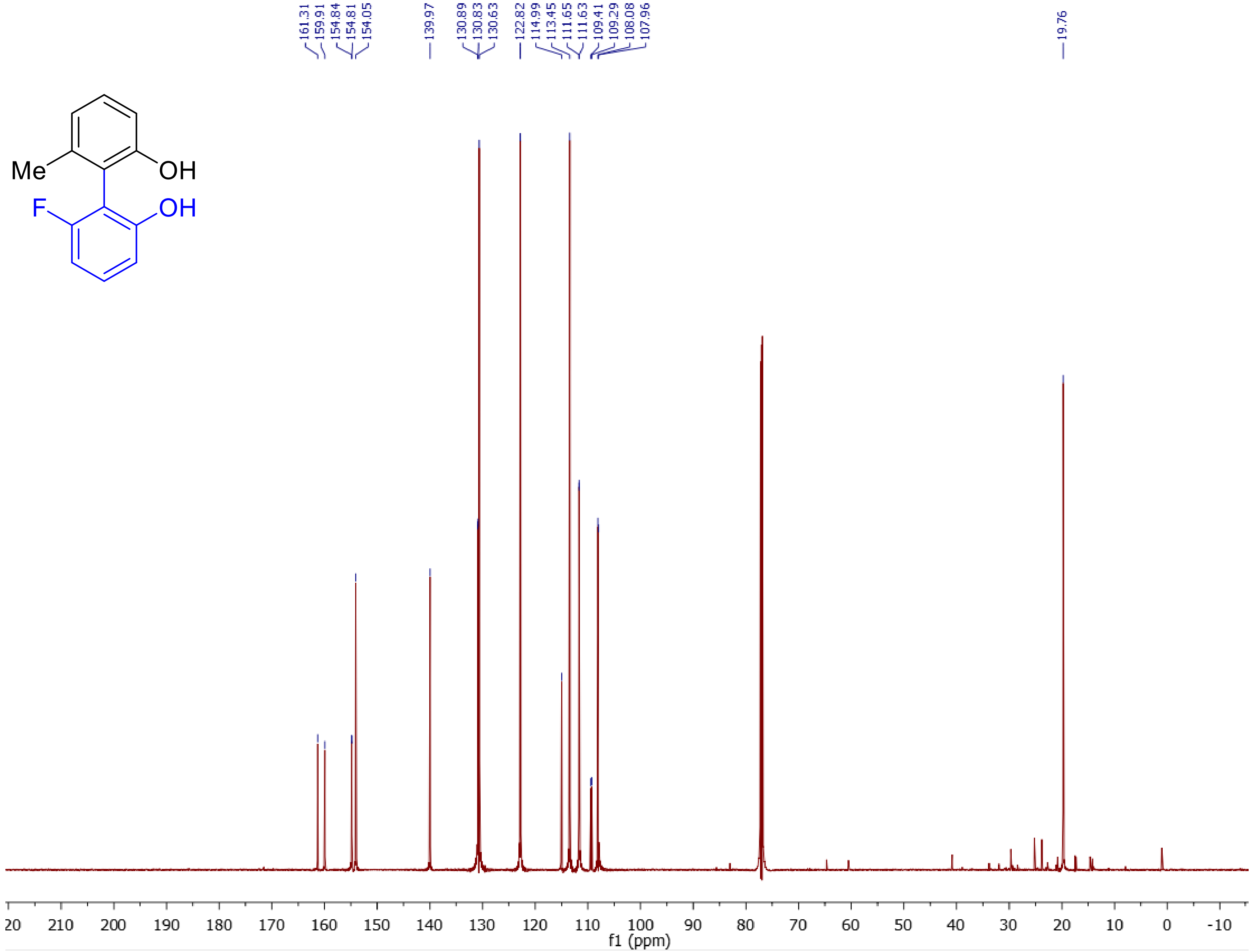

^{19}F-NMR ($CDCl_3$): (R)-6-fluoro-6'-methyl-[1,1'-biphenyl]-2,2'-diol

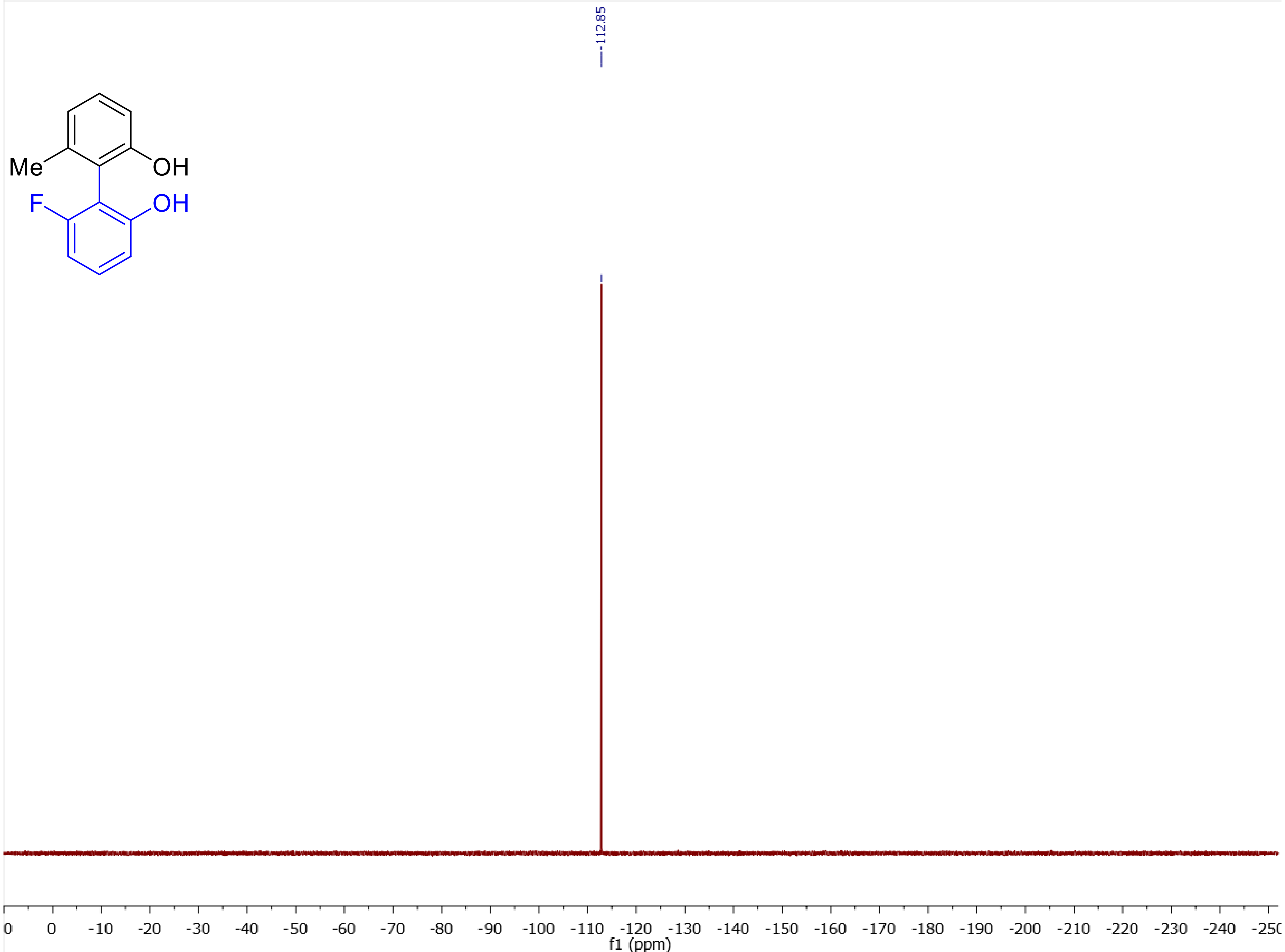

2'-(dicyclohexylphosphaneyl)-2,6-dimethoxy-[1,1'-biphenyl]-3-sulfonic acid

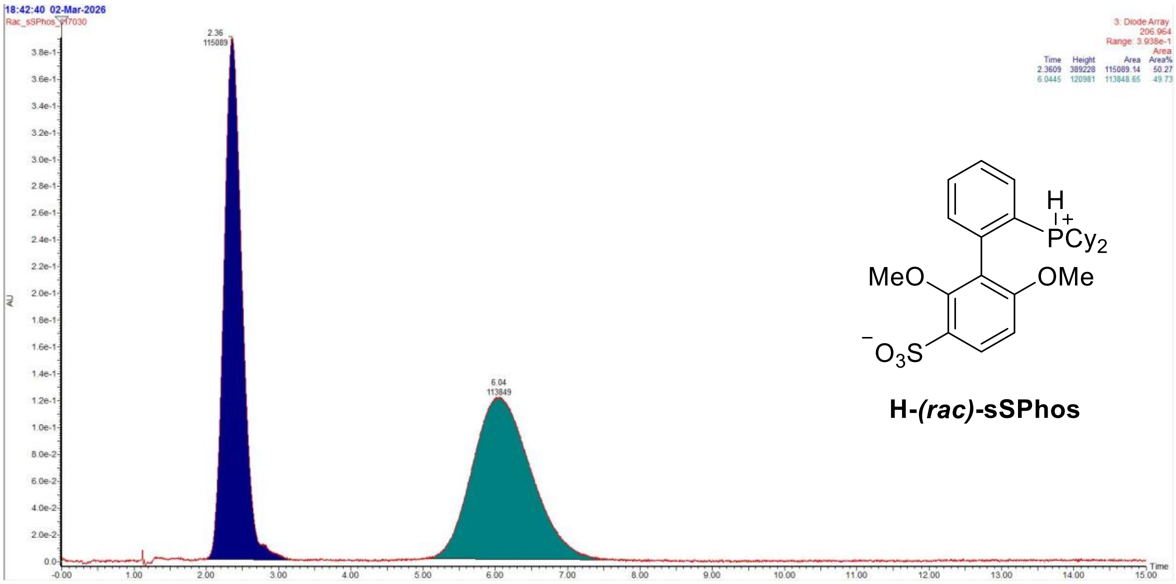

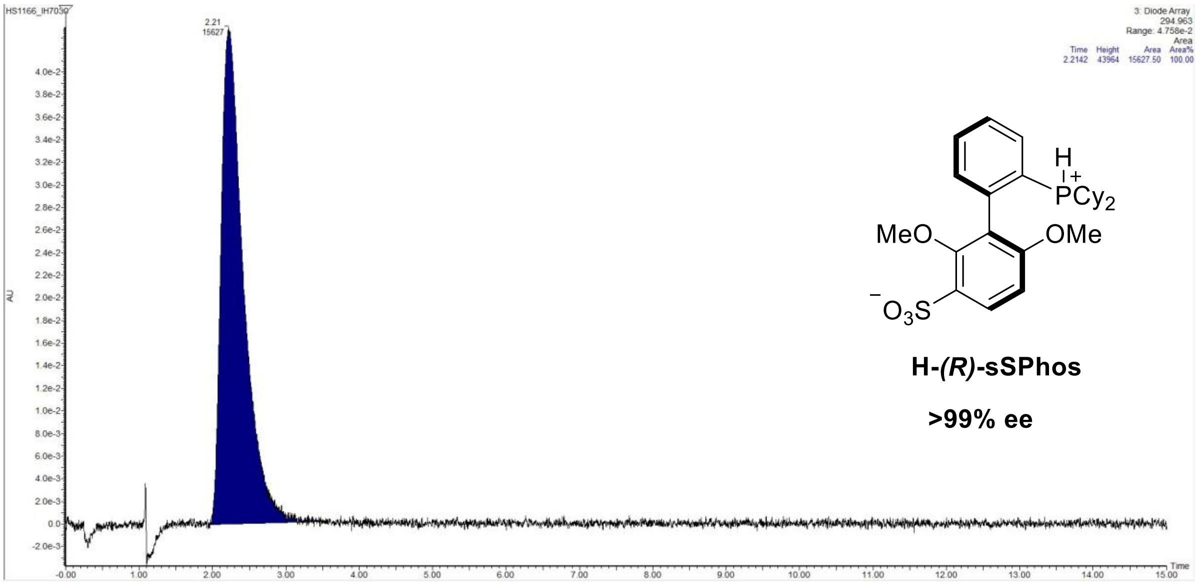

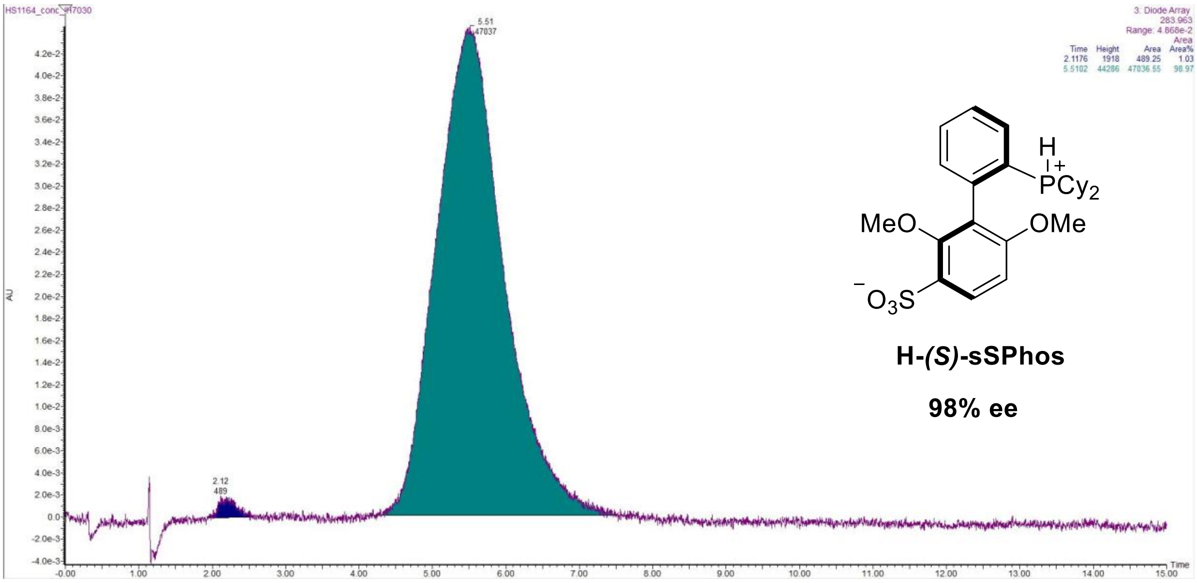

6-fluoro-6'-methyl-[1,1'-biphenyl]-2,2'-diol

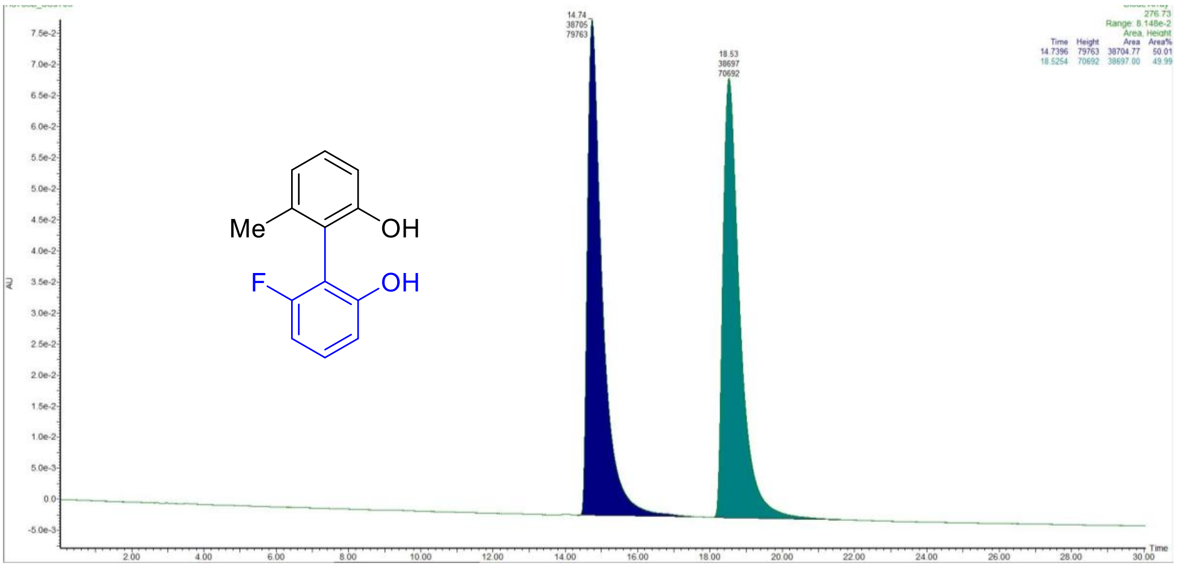

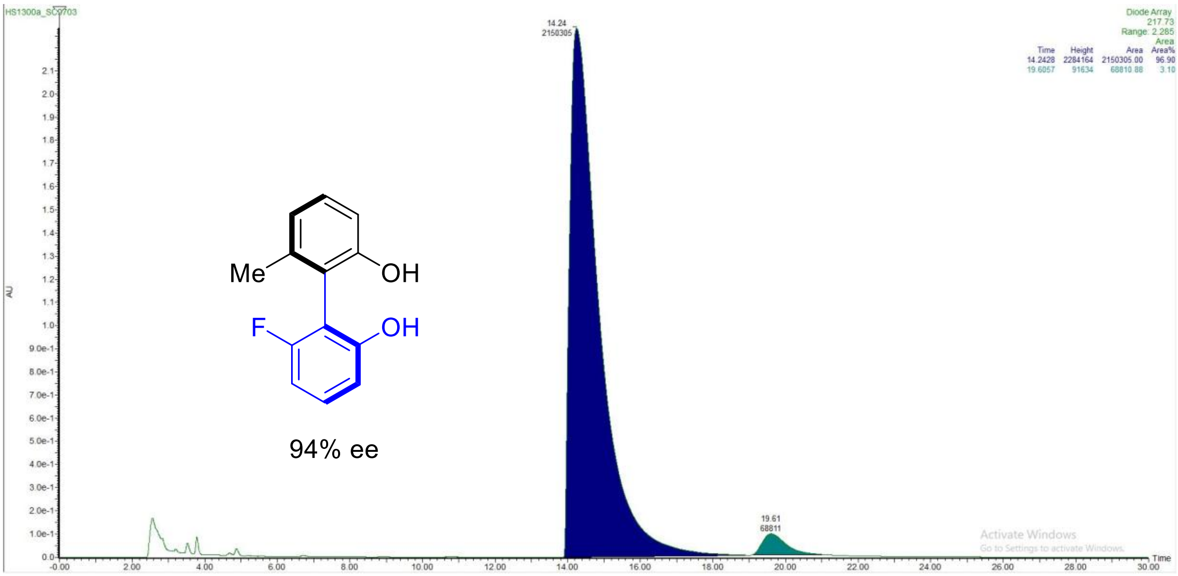

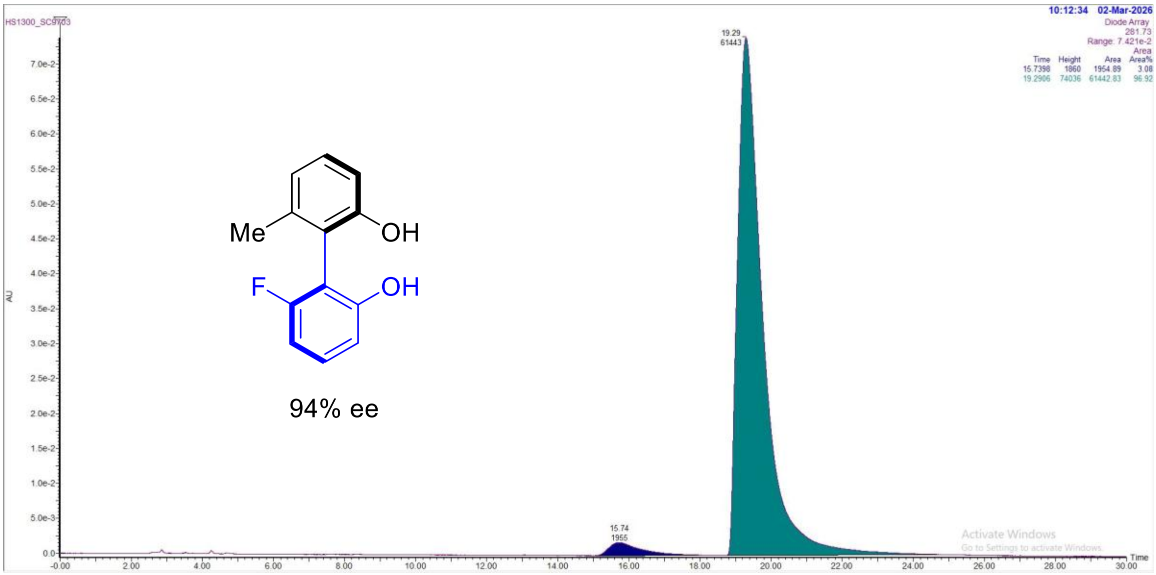

XRPD1 - Crystalline material directly after second recrystallisation

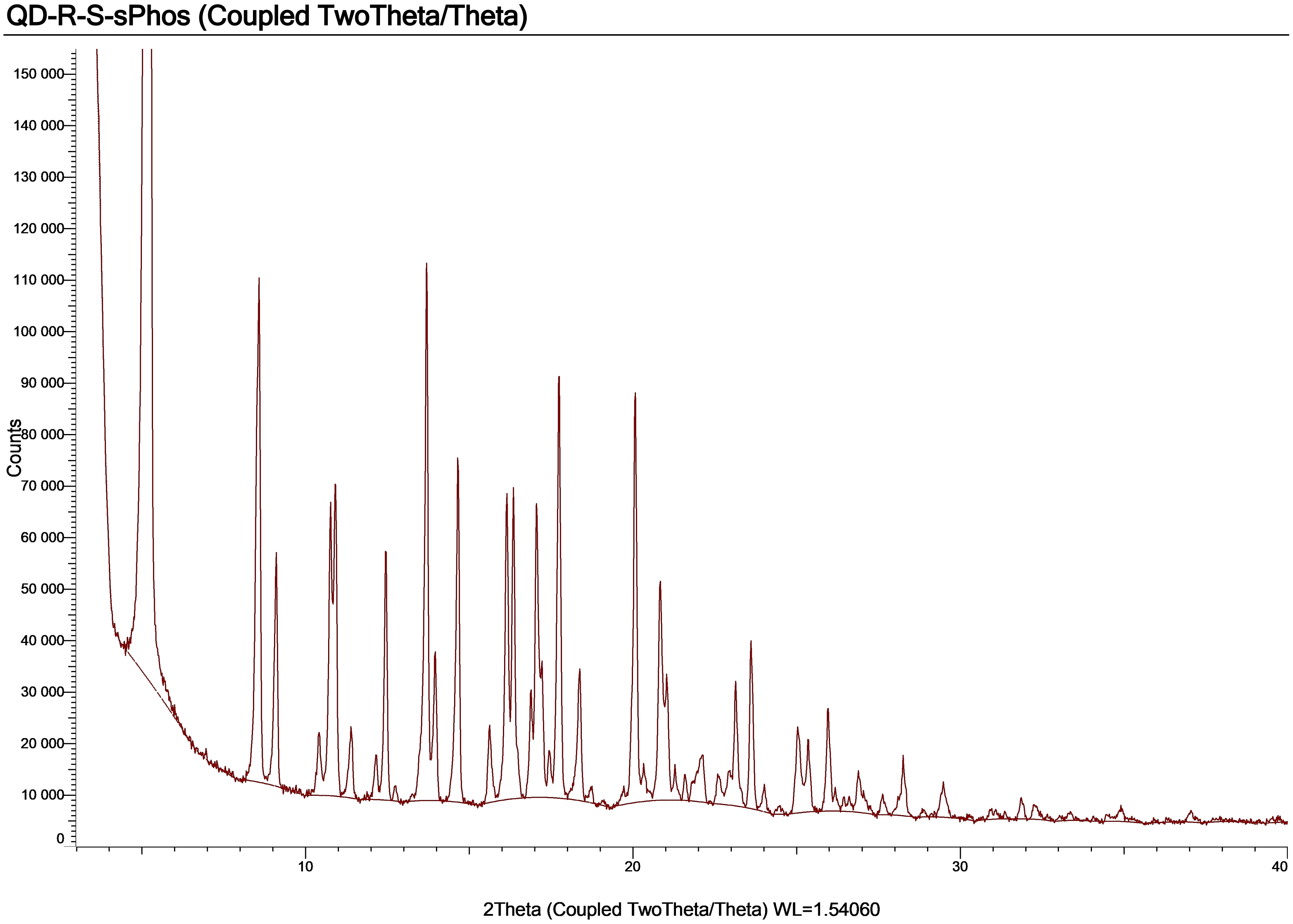

XRPD2 - Amorphous material after dissolving in methanol and evaporating

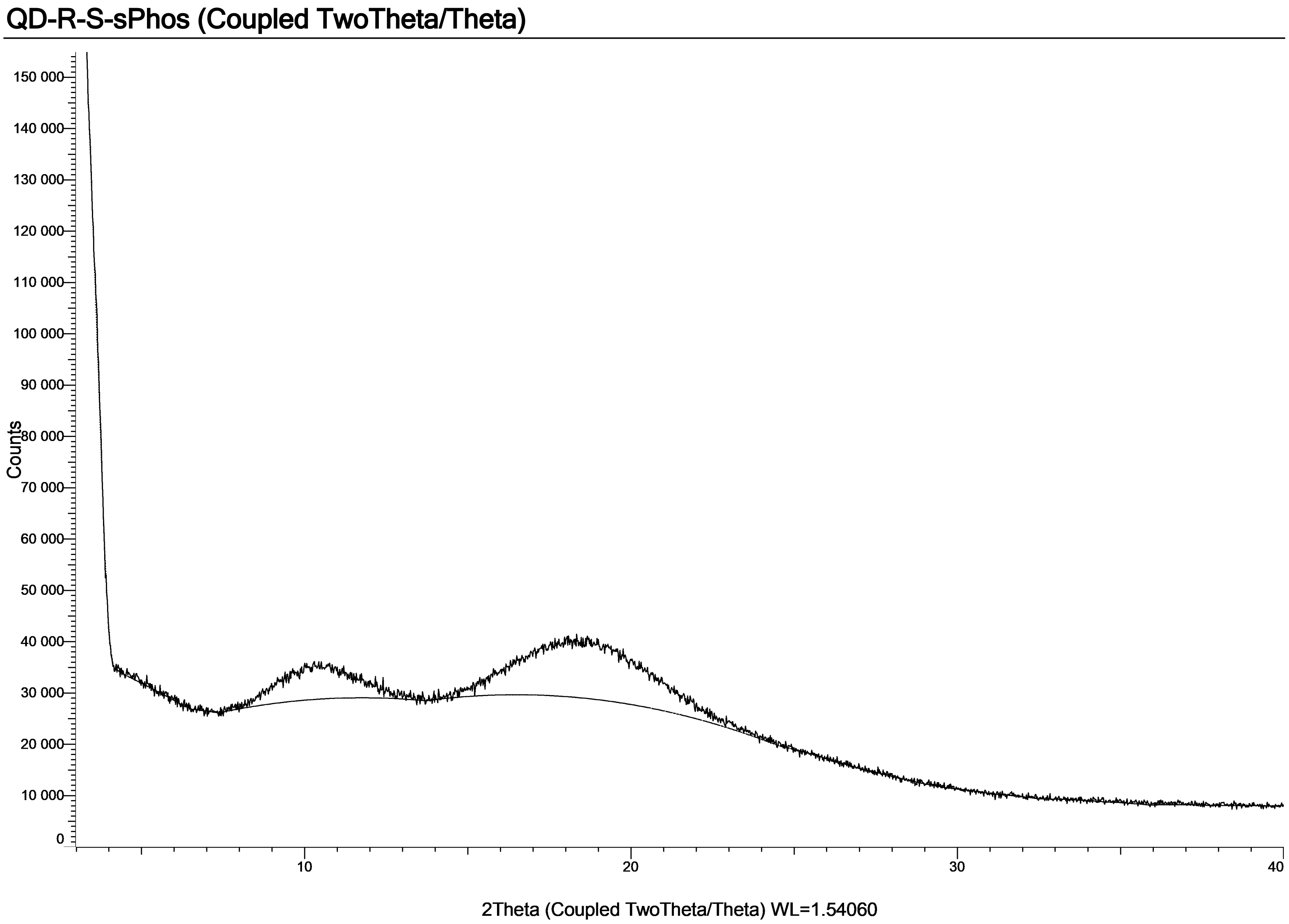

Supplement: Supplementary file 1 [file op6c00142_si_001.pdf]
